# The mRNACalc webserver accounts for the N1-methylpseudouridine hypochromicity to enable precise nucleoside-modified mRNA quantification

**DOI:** 10.1016/j.omtn.2024.102171

**Published:** 2024-03-11

**Authors:** Esteban Finol, Sarah E. Krul, Sean J. Hoehn, Xudong Lyu, Carlos E. Crespo-Hernández

**Affiliations:** 1 Programme in Emerging Infectious Diseases, Duke-NUS Medical School, National University of Singapore, Singapore 169857, Singapore; 2 Department of Chemistry, Case Western Reserve University, Cleveland, OH 44106, USA

**Keywords:** MT: Bioinformatics, N1-methylpseudouridine, pseudouridine, modified-nucleoside, mRNA, UV absorption

## Abstract

Nucleoside-modified messenger RNA (mRNA) technologies necessarily incorporate N1-methylpseudouridine into the mRNA molecules to prevent the over-stimulation of cytoplasmic RNA sensors. Despite this modification, mRNA concentrations remain mostly determined through the measurement of UV absorbance at 260 nm wavelength (A_260_). Herein, we report that the N1-methylpseudouridine absorbs approximately 40% less UV light at 260 nm than uridine, and its incorporation into mRNAs leads to the under-estimation of nucleoside-modified mRNA concentrations, with 5%–15% error, in an mRNA-sequence-dependent manner. We therefore examined the RNA quantification methods and developed the mRNACalc webserver. It accounts for the molar absorption coefficient of modified nucleotides at 260 nm wavelength, the RNA composition of the mRNA, and the A_260_ of the mRNA sample to enable accurate quantification of nucleoside-modified mRNAs.

## Graphical abstract

**Figure:**
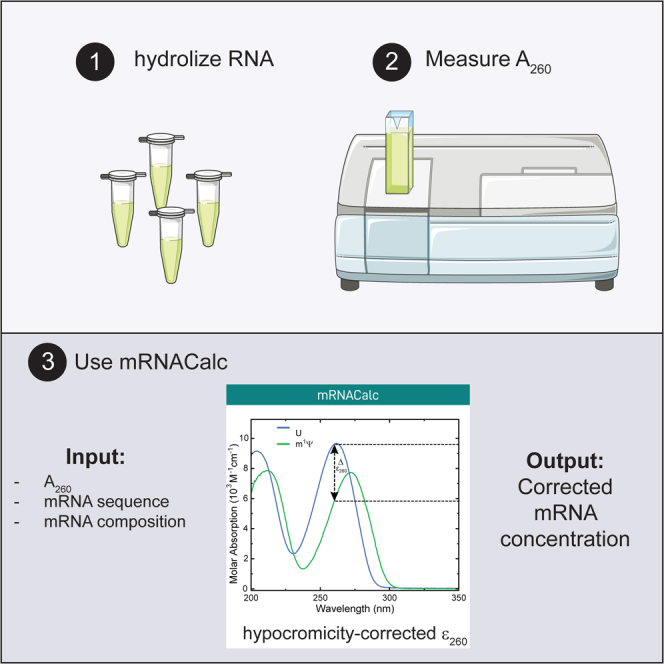

## Teaser

Finol and colleagues observed that the N1-methylpseudouridine (m^1^Ψ), which is incorporated in mRNA technologies, absorbs approximately 40% less UV light than uridine, affecting the traditional methods for mRNA concentration determination. They revisited these methods and developed a software that corrects for the m^1^Ψ hypochromicity, thus enabling accurate nucleoside-modified mRNA quantification.

## Introduction

The therapeutic use of messenger RNA (mRNA) has sparked great optimism in the development of novel vaccines and therapeutics against a myriad of infectious or as-yet-incurable diseases.^1^ The mRNA technology enables the production of antigenic, functional, and/or therapeutic proteins by introducing mRNA into the human body and cells.^2^ Since mRNAs act in the cytoplasm transiently, they do not bear any risk of integration into the host cell genome. Most importantly, the mRNA technology enables rapid, cost-efficient, and scalable production, which is free of cellular (cell cultures) or animal materials.^3^ Thus, mRNA technologies facilitate manufacturing and allow for a rapid response to emerging infectious diseases, as emphatically underscored by the rapid rollout of COVID-19 mRNA vaccines in many parts of the world. Modified nucleosides, such as pseudouridine (Ψ), N1-methylpseudouridine (m^1^Ψ), and 5-methylcytidine (m^5^C), are often incorporated into the mRNA molecules. Such modifications reduce stimulation of cytoplasmic RNA sensors, such as Toll-like receptors 3 and 7, for improved safety profiles and enhanced mRNA translation.^4^^,^^5^ However, how modified nucleosides affect mRNA concentration measurements and potentially confound preclinical dosing, efficacy, and toxicology studies, which could make or break further clinical development of any therapeutic, remains undefined.

The determination of RNA concentration often relies on measurements of its UV absorbance at 260 nm wavelength (A_260_) and the implementation of the Beer-Lambert law.^6^ The accuracy of these measurements is scattered by the variable hypochromicity of RNA due to its sequence-dependent folding. The molar absorption coefficient (MAC, or extinction coefficient [ε]) of a folded RNA at 260 nm (ε_260_) is reduced as compared to its unfolded state.^7^ This difference is buffer and concentration dependent and arises from changes in the chemical environment of the nucleobases—the main chromophore—due to base pairing, stacking, intermolecular interactions, and other conformational changes. Considering these variabilities, a rough estimation for the MAC_260_ of any single-stranded RNA (ssRNA), 40 μg/mL per absorbance unit, is extensively used, and its associated ±10%–20% error in the estimation of RNA concentration is widely accepted.^6^ This error range may suffice to assess dose response for mRNA therapeutics across several orders of magnitude *in cellula* or in *in vivo* experiments, yet it would be valuable to know concentrations at higher accuracy for the development of mRNA technologies. Our particular concern is in measurements of self-amplifying RNAs (saRNAs) and nucleoside-modified mRNAs. The logarithmic amplification of saRNA can convert a 20% accepted error in RNA concentration into several-fold differences in dose response between one experiment and subsequent replicates. The chemical modifications on the nucleobases of mRNA can also induce profound changes in the mRNA MAC hindering the accurate quantification of nucleoside-modified mRNA concentrations.

To attain greater accuracy in RNA quantification, RNA molecules are hydrolyzed prior to UV absorbance determination using a combination of thermal and alkaline hydrolysis.^6^^,^^8^ The RNA hydrolysis shifts the hypochromic folded state of the RNA to the hyperchromic state of the single monophosphate nucleotides.^9^ Since the precise MAC of the four standard nucleotides in aqueous buffered solution is known, the molar absorption of any hydrolyzed mRNA can be calculated as the sum of the molar absorption of its nucleotide compositions. Thus, upon the A_260_ determination, the RNA concentration can be quantified with an error of ∼4% using these methods.^6^ The incorporation of modified nucleosides can alter the RNA molar absorption and increase the error of the measurements in an RNA-sequence-dependent manner. Other non-UV-spectroscopic methods relying on the unspecific RNA binding of fluorophores for the determination of RNA concentration may help to overcome any change in the MAC of modified nucleoside mRNA. However, the impact of RNA modifications on the binding affinity of these fluorophores also remains unknown.

Herein, we report our effort to revisit and determine the MAC of modified nucleosides (Ψ, m^1^Ψ, and m^5^C). We also examined three different methods for RNA hydrolysis and provided them along with the mRNACalc webserver. This web tool incorporates the most recently revised MAC_260_ for standard, modified, and mRNA capping nucleosides, allowing the accurate determination of standard and nucleoside-modified mRNAs using UV spectroscopy.

## Results

**Figure 1:**
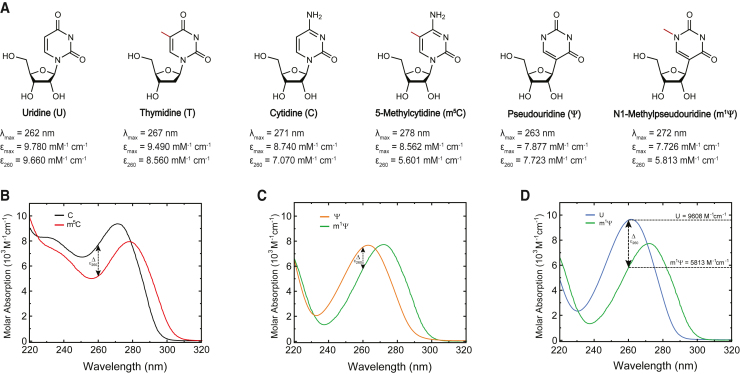
The nucleobase methylation and its bathochromic effect on the UV molar absorption spectra of pyrimidines (A) Skeletal formula of uridine, thymidine, cytidine, 5-methylcytidine, pseudouridine, and N1-methylpseudouridine. The methyl substituents are highlighted in red. These λ_max_, ε_max_, and ε_260_ values are implemented in the mRNACalc webserver. The source of these values is provided in the supplemental information. (B) Steady-state absorption spectra of cytidine (black line) and 5-methylcytidine (red line) at pH 7.4. (C) Steady-state absorption spectra of pseudouridine (orange line) and N1-methylpseudouridine (green line) at pH 7.4. (D) Steady-state absorption spectra of uridine (light blue line) and N1-methylpseudouridine (green line) at pH 7.4. The ε_260_ for U and m^1^Ψ are shown.

To assess the impact of chemical modifications on the spectrophotometric parameters of pyrimidine nucleosides for mRNA quantification, we determined and compared the molar UV absorption curves of standard nucleosides (U and C) and the modified nucleosides that have recently been employed in nucleoside-modified mRNA technologies (Ψ, m^1^Ψ, and m^5^C). For the cytidine-to-m^5^C comparisons, a shift of +7 nm in the peak maximum (Δλ_max_) was observed with a 20.8% reduction in the ε_260_ for the m^5^C nucleoside (Figures 1A and 1B). For the Ψ and m^1^Ψ curves, a similar shift was detected (Δλ_max_ = +9 nm in m^1^Ψ; Figure 1C), with a reduced molar absorption at 260 nm for m^1^Ψ (Δε_260_ = −22.8%). More importantly, m^1^Ψ is hypochromic as compared to uridine at λ_max_ (Δε_max_ = −21%), and, due to the λ_max_ shift, m^1^Ψ absorbs 39.8% less than uridine at 260 nm (Figure 1D), suggesting that m^1^Ψ-incorporated mRNAs can have reduced MACs.

**Figure 2:**
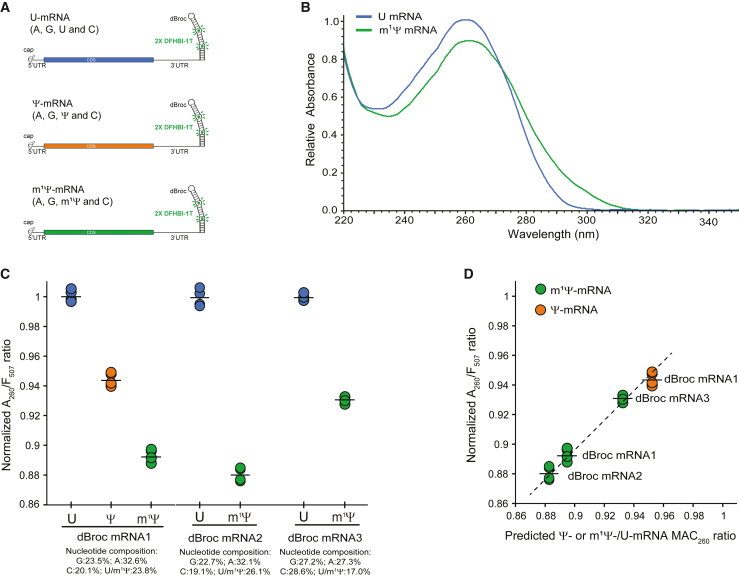
The hypochromicity of nucleoside-modified mRNA can be predicted from their nucleoside composition (A) Schematic representation of the mRNAs that were designed to determine the normalized A_260_/F_507_ values. (B) Relative UV absorption curves from mRNAs with uridine or N1-methylpseudouridine nucleosides. They were normalized to the corresponding F_507_ values and plotted relative to the peak maximum of the U-mRNA. (C) The normalized A_260_/F_507_ values from five replicates of the U-, Ψ-, and m^1^Ψ-mRNAs are shown for dBroc-mRNA1. Similar measurements in two additional U- and m^1^Ψ-mRNAs are shown. The black lines correspond to the average absorbance. Values are relative to the average absorbance of the U-mRNA. All comparisons of the mean relative A_260_/F_507_ values were significant (t test; p < 0.005). (D) The normalized A_260_/F_507_ values in (C) were plotted against their predicted hypochromicity using the mRNACalc software.

To assess whether the complete U-to-m^1^Ψ substitution alters the UV absorbance of an mRNA, the same mRNA was transcribed using either U, Ψ, or m^1^Ψ. These mRNAs also encoded a dimeric-Broccoli (dBroc) aptamer in their 3′ untranslated regions (UTRs) (Figure 2A). Once the DFHBI-1T fluorophore was bound to the G-quadruplex in the Broccoli aptamer, the mRNA emitted green light upon excitation.^10^ We also confirmed that the brightness, melting point, and affinity of the DFHBI-1T-Broccoli complex are not significantly perturbed by the U-to-Ψ or U-to-m^1^Ψ substitution (Table S1; Figure S1). After normalizing the UV absorbance (A_260_) of each mRNA by its corresponding fluorescence (F_507_), it was observed that, in practice, the relative UV absorbance of the nucleoside-modified mRNA was significantly reduced as compared to the standard mRNA (ΔΑ_260_ = −10.6%; Figures 2B and 2C). This hypochromicity was also independently observed in two additional mRNAs with either higher or lower m^1^Ψ composition (ΔΑ_260_ = −11.8% and −6.7%, respectively, in Figure 2C). These findings confirmed that m^1^Ψ-mRNAs are hypochromic and their hypochromicity is dependent on the nucleoside composition. To correct for the observed hypochromicity in nucleoside-modified mRNA, we built the mRNACalc software, which calculates the expected MAC_260_ of a hydrolyzed mRNA. It considers its nucleotide composition and the MAC of standard and modified nucleosides, including the nucleosides in the mRNA cap (documentation in the supplemental information and Tables S2–S6). We used this software to predict MAC_260_ for the different U-, Ψ-, and m^1^Ψ-dBroc-mRNAs in Figure 2C and plotted their Ψ-/U-mRNA and m^1^Ψ-/U-mRNA MAC_260_ ratios against the experimentally determined normalized A_260_/F_507_ ratio (Figure 2D). The observed linearity in this graph corresponds to the expected linearity in the Beer-Lambert law for standard and modified nucleosides and its implementation in ssRNAs, such as mRNA.

**Figure 3:**
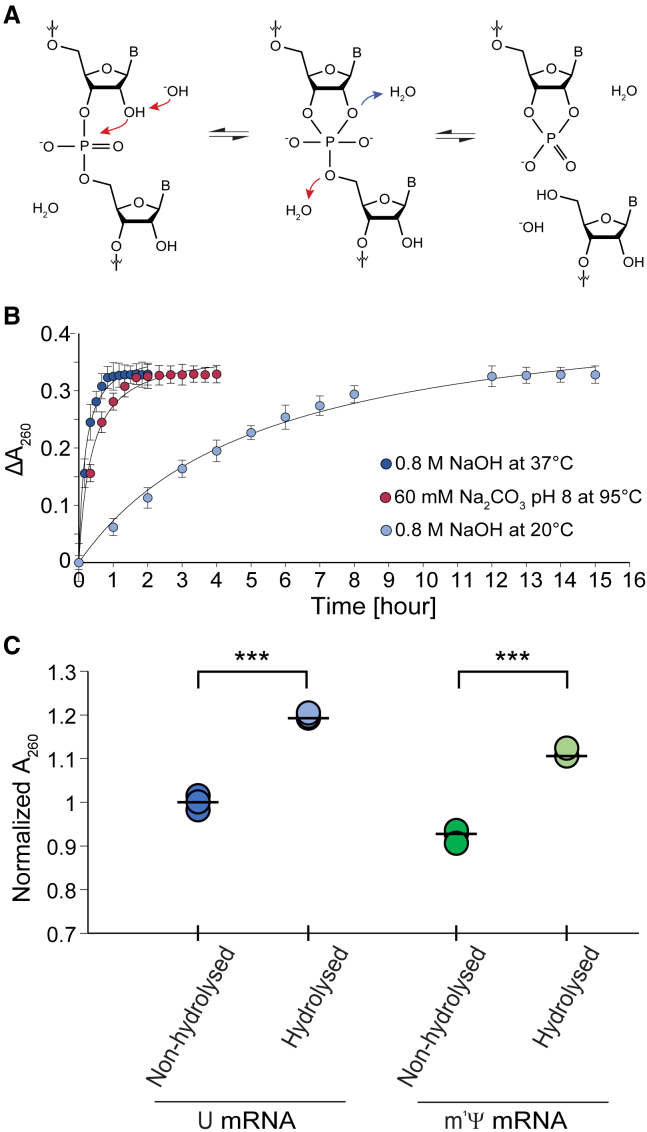
RNA hydrolysis is essential for the determination of mRNA concentrations (A) Alkali-promoted transesterification allows RNA hydrolysis and mRNA quantification. Under alkaline conditions, the reactive -OH triggers the nucleophilic attack of the 2′-OH on the 3′,5′-phosphodiester linkage, converting the ground-state configuration of RNA into a penta-coordinated intermediate and leading to a 2′3′-cyclic phosphodiester. This cyclic form is then known to form 3′ and 2′ monophosphate nucleotides (data not shown). (B) Thermal and/or alkaline hydrolysis of RNA over time. Yeast RNA was hydrolyzed using three different previously described methods and the ΔA_260_ was determined using a UV spectrophotometer at different intervals. For expedited RNA hydrolysis (1 or 2 h incubation), a combination of thermal and alkaline hydrolyses can be used (dark blue dots, 0.8 M NaOH at 37°C; red dots, 0.5 M Na_2_CO_3_ [pH 8] at 95°C). For overnight incubation, alkaline hydrolysis suffices (light blue dots, 0.8 M NaOH at 20°C, the last four measurements were performed after an overnight incubation). Dots indicate the mean value of three measurements. Error bars correspond to standard deviations. (C) Hydrolysis of U-mRNA and m^1^Ψ-mRNA using 0.8 M NaOH at 37°C increases the UV absorption of mRNA. This mRNA corresponds to dBroc-mRNA3 in (C). A_260_ values are normalized to the mean A_260_ values of the non-hydrolyzed U-mRNA.

To enable accurate measurement of nucleoside-modified mRNA, we also assessed different RNA hydrolysis methods. The modern analytical use of alkaline hydrolysis of RNA has been known since 1922, when Steudel and Peiser demonstrated that 1 M NaOH hydrolyzed yeast RNA whereas thymus DNA resisted the NaOH hydrolysis.^11^ The alkali-promoted transesterification of RNA occurs due to the nucleophilic attack of the 2′-OH in the ribose to the 3′,5′-phosphodiester bond, explaining the alkali resistance of the 2′-deoxyribonucleotides (Figure 3A).^12^ This reaction is further catalyzed with the introduction of heat. However, the combination of thermal and alkaline hydrolysis, e.g., 1 M NaOH at 95°C, also catalyzes the deamination of cytosine to uridine in a small percentage of residues.^13^^,^^14^ Thus, a compromise between the two methods is often applied. In our hands, three of such protocols showed a similar increase in A_260_ upon hydrolysis of yeast RNA—a historical standard sample for these methods (Figure 3B). One of these methods (0.8 M NaOH at 37°C) was also applied on U- and m^1^Ψ-mRNAs (Figure 3C), and the use of RNA hydrolysis indeed increased the A_260_ of both types of mRNA, confirming the importance of performing RNA hydrolysis to remove the effect of RNA folding on the mRNA UV absorption and therefore allow a more accurate determination of mRNA concentrations. We also applied the RNA hydrolysis methods on U- and m^1^Ψ-mRNAs and determined their concentration by measuring their A260 and using the mRNACalc software to correct for hypochromicity. The concentration of these mRNAs was then reassessed by performing direct A_260_ measurements, without prior RNA hydrolysis and implementing the extensively used MAC_260_ for ssRNA (40 μg/mL per absorbance unit), or by using a commercially available fluorescence-based assay. We could observe that both methods differentially estimated the nucleoside-modified and standard mRNA concentrations, with an underestimation of the m^1^Ψ-mRNA concentration (Figure S2).

## Discussion

Ψ is an isomer of uridine—the standard nucleoside in RNA. Ψ, as opposed to other nucleosides, is a carbon-carbon ribofuranosyl nucleoside, i.e., the uracil nucleobase is linked to the ribose through its fifth carbon instead of an N1 linkage.^15^ This unique arrangement places the N1 imino group toward the so-called “C-H” edge of the pyrimidine ring and confers additional properties to this edge in Ψ. This imino hydrogen proton is susceptible to hydrogen bonding, chemical exchange, and chemical modifications such as N1 methylation. Thus, the m^1^Ψ, as well as the m^5^C, represents a modification of the C-H edge of the pyrimidine nucleobase. The influence of a 5-methyl substituent on the UV molar absorption of pyrimidine rings has been well known since the 1940s, when Sister Miriam Michael Stimson showed that a similar 5-methyl modification also differentiates uridine from thymidine and provokes a subtle reduction in molar absorbance (ΔMAC_max_ = −3%) and a shift of the peak maximum (Δλ_max_ = +5 nm) to a longer wavelength—a bathochromic shift.^16^^,^^17^^,^^18^^,^^19^ In combination, these two effects provoke a substantial MAC_260_ reduction for the thymidine nucleoside (ΔMAC_260_ = −11.4%). In our study, similar differences were observed for the C-to-m^5^C and Ψ-to-m^1^Ψ comparisons, with a more pronounced MAC_260_ difference for the U-to-m^1^Ψ comparison. Thus, the substitution of uridine by m^1^Ψ in mRNA technologies can substantially modify the spectrophotometric properties of the mRNA.

In principle, the modified nucleosides may also promote mRNA folding and reduce its UV absorption. This is particularly relevant for the Ψ modification. Its N1 hydrogen can engage in additional hydrogen bonds, promoting and stabilizing RNA folding. For instance, the U-to-Ψ substitution in tRNA stabilizes the folded structure that is essential for translation.^20^ However, the m^1^Ψ nucleobase lacks this additional hydrogen bonding capability, and it is expected to have little or no effect on the RNA folding of less structured RNA molecules such as mRNAs. Considering that both Ψ- and m^1^Ψ-mRNAs followed the anticipated hypochromicity that is associated with the modified nucleosides’ hypochromicity at 260 nm wavelength (Figure 1) and their abundance in the mRNA (Figure 2C), rather than the expected distinct contribution of Ψ and m^1^Ψ to RNA folding, we can conclude that the observed reduction in the UV absorption of nucleoside-modified mRNA is mainly determined by the nucleobase composition and the intrinsic MAC of the nucleosides in the purified mRNAs. Importantly, the UV absorption spectrum of the m^1^Ψ-mRNA also depicted a broad absorption peak and a bathochromic shift, which brings about additional implications for the assessment of the RNA sample purity (Figure 2B; supplemental information). These findings indicate that for accurate determination of nucleoside-modified mRNA concentrations and proper interpretation of dose-ranging preclinical studies, the reported UV spectroscopic differences must be accounted for. Otherwise, nucleoside-modified mRNA concentrations may be underestimated by 5%–15% depending on the proportion of m^1^Ψ in the mRNA composition.

**Figure 4:**
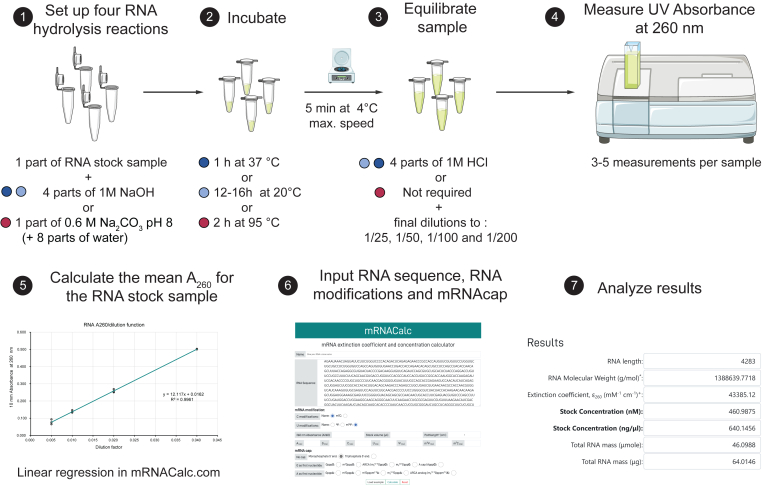
Experimental workflow for the determination of RNA concentration using the mRNACalc webserver The colored dots refer to the different RNA hydrolysis methods in Figure 3B.

Considering that traditional methods underestimate the nucleoside-modified mRNA concentrations and to ease the implementation of the reported UV absorption parameters, we provide the mRNACalc software as an open-source webserver to calculate the MAC_260_ for nucleoside-modified mRNAs. It accounts for the hypochromicity of modified nucleosides as well as for the nucleoside composition of the mRNA, including the mRNA cap. Once the RNA sequence, the A_260_, and the RNA stock volume values are provided as input, the mRNACalc webserver calculates the RNA stock concentration in nM and ng/μL and the total RNA mass in μmole and μg. The webserver also includes the revisited experimental protocols and a workflow that implements a linear regression model from multiple measurements at serial dilutions (Figure 4). This workflow aims at reducing the impact of sample handling variation. Hence, the mRNACalc webserver represents a freely available and all-inclusive tool for the determination of nucleoside-modified mRNA concentrations using UV spectroscopy.

## Materials and methods

### The Beer-Lambert experiments

Ψ (≥98% purity), m^5^C (≥99% purity), cytidine (99% purity), and uridine (99% purity) were purchased from Sigma-Aldrich. m^1^Ψ (>95% purity) was purchased from Biosynth Carbosynth. They were used as received. Phosphate buffer solutions with a total phosphate concentration of 16 mM from monosodium and disodium phosphate salts dissociated in ultrapure water (Millipore) were freshly prepared on the day of each experiment. The pH of the solution was adjusted using 0.1 M solutions of NaOH and HCl to the desired pH of 7.4 (±0.1 pH units). Steady-state absorption was recorded using a Cary 100 spectrometer. Serial dilutions of known concentration were carried out such that the absorbance reading at the respective lambda maximum (local maximum absorbance) remained below 1.0, within the linear range of the instrument. The MACs were experimentally determined using the slope from the linear regression from plotting absorbance versus concentration. The correlation constant for the linear regression analysis of the Beer-Lambert’s law data for determining molar absorption constants was >0.9999, showing a strong linear relationship.

### mRNA *in vitro* transcription and purification

The plasmid DNA template (pUCIDT plasmid) was grown in DH5 alpha *E. coli* (New England Biolabs) in 300 mL Luria-Bertani broth supplemented with kanamycin (50 μg/mL), and a maxi preparation was performed using the QIAGEN Plasmid Plus Maxi Kit following manufacturer instructions. The plasmid encoded a T7 promoter followed by the mCherry gene with a degradation tag (1,449 nucleotides) plus the 3′ and 5′ UTRs of the BNT162b2 mRNA vaccine (541 nucleotides). The double broccoli aptamer was encoded within the poly-adenine region in the 3′ UTR. The plasmid was linearized by EcoRV restriction enzyme digestion at the end of the 3′ UTR.

A standard T7 transcription reaction included 30 mM Tris-HCl (pH 7.9), 2 mM spermidine, 30 mM MgCl_2_, 5 mM NaCl, 10 mM DTT, 50 μg/mL BSA (New England Biolabs), 0.005% Triton X-100, 2% polyethylene glycol (PEG8000), 5 mM of each triphosphate ribonucleotide (standard nucleotides were purchased from Jena Bioscience GmBH and Ψ and m^1^Ψ from BOC Sciences), 2 μM linearized plasmid DNA template, 3.5 μM T7 RNA polymerase (in-house produced and purified), and 0.0025 units of *E. coli* inorganic PPase (New England Biolabs). All reagents were purchased from Sigma-Aldrich unless otherwise stated. The reactions were incubated at 37°C for 2.5 h and stopped by the addition of 500 mM EDTA (pH 8) to a final concentration of 35 mM.

The mRNA was purified using anion-exchange chromatography. A PRP-X600 anion-exchange column (Hamilton Company) was equilibrated in buffer A (85:15 100 mM Tris [pH 8]/acetonitrile). RNA samples were loaded onto the column at a flow rate of 3 mL/min and eluted with a 40 min gradient of 0%–40% buffer B (85:15 100 mM Tris, 2.5 M LiCl [pH 8]/acetonitrile). Fractions containing the mRNA were collected, and the mRNA molecules were precipitated using standard butanol extraction.^21^ The purity of the mRNA preparation was assessed using high-resolution automated electrophoresis in the Agilent 2100 Bioanalyzer system using the Bioanalyzer RNA 6000 pico assay (Agilent Technologies).

### Determination of the mRNA UV absorption spectrum

To determine the UV absorption spectrum of mRNAs, the mRNA stocks were diluted to approximately 25 nM into a buffer containing 40 mM HEPES (pH 7.4), 5 mM MgCl_2_, and 100 mM KCl to a final volume of 2 mL. Five independent mRNA samples were prepared per mRNA set (U-, Ψ-, and m^1^Ψ-mRNAs). The UV absorption spectra were recorded for each mRNA sample using a UV-3600i plus UV-visible (UV-vis) spectrophotometer (Shimadzu Corp.).

### Excitation-emission experiments on the DFHBI-1T-bound mRNAs

After UV absorption determination, the mRNA samples were bound to the DFHBI-1T fluorophore by adding 100 μM DFHBI-1T and 100% DMSO to a 500 nM concentration into the 2 mL mRNA samples. Fluorescence was measured with a Fluorolog-3 spectrofluorometer (Horiba Scientific) using the excitation and emission wavelengths commonly used for DFHBI-1T (excitation: 472 nm, emission: 507 nm).^10^

### Determination of the relative UV absorbance (A_260_)

The A_260_/F_507_ ratios were calculated for each mRNA sample. The mean A_260_/F_507_ values for U-, Ψ-, and m^1^Ψ-mRNAs were calculated. The A_260_/F_507_ values of each sample were normalized using the mean A_260_/F_507_ value from the U-mRNA as reference, and they were plotted in a dot plot. The t tests were applied to compare the mean A_260_/F_507_ values across each pair of mRNA sets using a p value of 0.005 as the cutoff of significance.

### Methods of RNA hydrolysis

Two methods of RNA hydrolysis were tested in this study. Torula yeast RNA was used as standard RNA sample (Sigma-Aldrich). The yeast RNA stock was prepared at 1,000 μg/μL in water. Thus, after 1/25 dilution, the UV absorbance of this RNA sample would be within the linear range of the instrument (UV-3600i plus UV-vis spectrophotometer, Shimadzu Corp.).

The most extensively used alkaline RNA hydrolysis method involves adding 1 part RNA and 4 parts 1 M NaOH and incubating them at 37°C for 1 h.^22^ To test this method, twelve yeast RNA samples were hydrolyzed. Every 10 min, a sample was neutralized with 4 parts 1 M HCl and diluted to 1/25 with 16 parts water. Three UV absorbance measurements were performed on every sample. Similarly, a room temperature variation of this method is often used for overnight RNA hydrolysis. Therefore, twelve RNA samples were hydrolyzed and incubated at 20°C for up to 15 h. Samples were neutralized and diluted hourly followed by three UV absorbance measurements.

A second method of thermal hydrolysis at neutral pH was also tested.^8^ To test this method, twelve yeast RNA samples hydrolyzed (1 part RNA in 9 parts 60 mM Na_2_CO_3_ [pH 8]) with an incubation of at 95°C for up to 2 h. Every 20 min, a sample was diluted to 1/25 with 15 parts water, and three UV absorption measurements were performed on every sample.

## Data and code availability

The data that support the findings of this study are available from the corresponding author upon reasonable request. The webserver is available at https://www.mrnacalc.com. The website is free and open to all users, and there is no login requirement. The HTML script for the mRNACalc webserver is available under a GNU general public license from https://github.com/estebanfbfc/mRNACalc. It can be downloaded free of charge and run locally without internet access.

## Supplemental information

Document S1. Supplemental materials and methods, Tables S1–S6, Figures S1, and S2

Document S2. Article plus supplemental information
