# Supplementary material for: The mRNACalc webserver accounts for the N1-methylpseudouridine hypochromicity to enable precise nucleoside-modified mRNA quantification
Source: Mol Ther Nucleic Acids. 2024 Mar 11;35(2):102171. doi: 10.1016/j.omtn.2024.102171 (PMC10973171; doi:10.1016/j.omtn.2024.102171)
Supplement: Document S1. Supplemental materials and methods, Tables S1–S6, Figures S1, and S2 [file mmc1.pdf]

OMTN, Volume *35*

Supplemental information

The mRNACalc webserver accounts for the N1-methylpseudouridine hypochromicity to enable precise nucleoside-modified mRNA quantification

Esteban Finol, Sarah E. Krul, Sean J. Hoehn, Xudong Lyu, and Carlos E. Crespo-Hernández

Supplemental Material

On the calculations and parameters in the mRNACalc webserver

- The mRNA molar absorption coefficient (ε) is calculated from the sum of the individual nucleotide extinction coefficients as determined by:

$$\varepsilon_{mRNA} = n_A\varepsilon_A + n_G\varepsilon_G + n_C\varepsilon_C \; or \; n_{m^5C}\varepsilon_{m^5C} + n_U\varepsilon_U \; or \; n_\Psi\varepsilon_\Psi \; or \; n_{m^1\Psi}\varepsilon_{m^1\Psi} + \varepsilon_{cap}$$

  Where n_N corresponds to the number of each type of nucleotide, N, in the mRNA and ε_N to the molar absorption coefficient for each type of nucleotide, including the capping nucleotide (ε_{cap}).
- The mRNA molecular weight is calculated as the sum of the nucleotide composition mass as RNA-incorporated monophosphate nucleotides.

$$MW_{mRNA} = n_A MW_A + n_G MW_G + n_C MW_C \; or \; n_{m^5C} MW_{m^5C} + n_U MW_U \; or \; n_\Psi MW_\Psi \; or \; n_{m^1\Psi} MW_{m^1\Psi} + MW_{cap}$$

- The mRNA molar concentration is calculated using the Beer-Lambert equation:

$$Concentration\ (M) = \frac{A260}{\varepsilon_{mRNA} * cm^{-1}}$$

  The mRNA molar concentration is presented in the nM and ng/μl scales in the webserver.

On the molar absorption coefficients of nucleosides/nucleotides

For standard nucleotides, the mRNACalc webserver implements the ε_{260} parameters in Table S2. The parameters from Cavaluzzi et al. were obtained after accurate measurements of nucleotides concentration using nuclear magnetic resonance spectroscopy.

For the modified nucleosides/nucleotides, we have determined and searched for ε_{max} and ε_{260} parameters in the literature and in the datasheet of Ψ, $m^1\Psi$, and m^5C manufacturers, which are summarized in Tables S3, S4 and S5.

Considering the extensive variability across the published and manufacturer-provided values for m^5C and Ψ, the mRNACalc webserver implements the average ε_{260} values. For $m^1\Psi$, the mRNACalc webserver implements the ε_{260} value that was obtained for this study, due to the limited number of previously reported values.

For the mRNA capping nucleotides, the mRNACalc webserver implements the ε_{260} values provided by the manufactures (Table S6). In few cases, the ε_{260} values were not available, the independent ε_{260} values of the two nucleotides were summed up.

Overall, these mRNA cap parameters were only implemented for completeness, and they can be considered as rough estimations, despite their contribution to an mRNA UV absorption is rather negligible.

Important note: The molar absorption parameters, herein compiled, correspond to either nucleosides or nucleotides in aqueous buffered solution (pH 7 – 8). Considering that the contribution of the phosphate group to the molar absorption of nucleotides is negligible, they have been considered for their implementation in the mRNACalc webserver indiscriminately.

On the purity of RNA samples:

The presence of impurities in nucleic acid samples is often assessed using the $A_{260:280}$ and $A_{260:230}$ ratios. For pure RNA the $A_{260:280}$ ratio is ~ 2.0. This ratio is commonly used to assess the amount of protein contamination, since proteins absorb at 280 nm. Similarly, the $A_{260:230}$ ratio for pure RNA is often slightly higher than the $A_{260:280}$ ratio, ranging from 2.0 to 2.2. Residual chemical contamination (phenol, butanol, carbohydrates, guanidine, and others) from the RNA purification method can increase the A_{230} and reduce the $A_{260:230}$ ratio.

From our experience, the assessment of purity for the $m^1\Psi$ modified mRNA samples requires shifting the wavelength for these ratios to $A_{264:284}$ and $A_{264:234}$ due to the bathochromic shift in the mRNA absorption curve (Figure S3). Which leads to:

- a reduced A_{260} due to the λ_{max} shift (λ_{max} at ~264 nm),
- an increased A_{230} due to the shift on the curve trough (λ_{min}) to ~234 nm,
- and an increased A_{280} due to the absorbance peak shift and broadening. The broadening arises due to the wider range of λ_{max} values in the RNA composition (standard mRNA= 252 to 263 nm, $m^1\Psi$ modified mRNA= 252 to 272 nm) as well as, due to the broader absorption peak of $m^1\Psi$ (as determined by the peak width at the trough level: Urd= 52.5 nm vs $m^1\Psi$= 57.0 nm).

Thus, $A_{264:284}$ and $A_{264:234}$ ratios should be interpreted in the same manner as the $A_{260:280}$ and $A_{260:230}$ ratios, respectively. Alternatively, the $A_{260:280}$ ratio can be accepted at 1.9 and the $A_{260:220}$ ratio can range between 1.9 to 2.1.

Importantly, m^5C-modified mRNA should show similar modifications in the UV molar absorption spectrum and the proposed shifted ratios may be applied as well.

Supplemental tables:

Table S1: photophysical and biochemical properties of mutated Broccoli−DFHBI-1T complexes.

Complex	λ_{max} (nm)	Relative brightness*	K_D (nM)⁺	T_m (°C)⁺
U-Broc−DFHBI-1T	507	------	360	48
U-Broc−DFHBI-1T	507	1.000 ± 0.002	379.6 ± 13.89	49.13 ± 0.13
Ψ-Broc-DFHBI-1T	507	1.005 ± 0.004	378.7 ± 8.11	49.46 ± 0.09
$m^1\Psi$-Broc-DFHBI-1T	507	1.004 ± 0.003	375.6 ± 8.17	49.23 ± 0.07

*Relative to the U-Broc-DFHBI-1T complex. Data are shown as mean ± SD.
⁺ Data are shown as fitted K_D ± Error of the fit or fitted T_m ± Error of the fit.

Table S2: Molar absorption coefficients of standard nucleosides as reported in Cavaluzzi et al.

Standard nucleosides	λ_{max} (nm)	ε_{max} ($mM^{-1}cm^{-1}$)	ε_{260} ($mM^{-1}cm^{-1}$)
Uridine	262	9.78	9.66
Uridine*	262	9.66	9.60
Thymidine	267	9.49	8.56
Cytidine	271	8.74	7.07
Cytidine*	271	9.34	7.67
Guanosine	252	14.09	12.08
Adenosine	259	15.04	15.02

Source: Cavaluzzi et al. [1] and *this study.

Table S3: Molar absorption coefficients of pseudouridine as reported in the literature and manufacturers' datasheets.

Source	λ_{max} (nm)	ε_{max} ($mM^{-1}cm^{-1}$)	ε_{260} ($mM^{-1}cm^{-1}$)
Basanta-Sanchez et al. [2]	262	7.583	7.492
Yu & Allen.[3]	263	7.5	Not provided
David & Allen. [4]	263	8.4	8.3
Shapiro & Chambers. [5]	262	7.9	Not provided
Michelson & Cohn. [6]	262	8.0	Not provided
Cohn. [7]	263	8.1	Not provided
Jena Biosciences	265	7.9	Not provided
Trilink Biotechnologies	262	7.546	Not provided
This study	263	7.677	7.527
Average	263	7.877	7.723⁺

⁺Average ε_{260} was calculated by multiplying the average ε_{max} by the observed $\varepsilon_{260/263}$ ratio.

Table S4: Molar absorption coefficients of N1-methylpseudouridine as reported in the literature and manufacturers' datasheets.

Source	λ_{max} (nm)	ε_{max} ($mM^{-1}cm^{-1}$)	ε_{260} ($mM^{-1}cm^{-1}$)
Roche	271	7.3	Not provided
Trilink Biotechnologies	271	8.877	Not provided
This study	272	7.726	5.813
Average	271	7.967	5.994^{+}

$^{+}$Average ε_{260} was calculated by multiplying the average ε_{max} by the observed $\varepsilon_{260/272}$ ratio.

Table S5: Molar absorption coefficients of 5-methylcytidine as reported in the literature and manufacturers' datasheets.

Source	λ_{max} (nm)	ε_{max} ($mM^{-1}cm^{-1}$)	ε_{260} ($mM^{-1}cm^{-1}$)
Szer. [8]	278.5	8.8	Not provided
Martínez-Fernández et al.[9]	278	8.92	Not provided
Fox et al.[10]	277.5	8.88	Not provided
Ma et al.[11]	278	8.871	Not provided
Fox & Shugar. [12]	276	8.05	Not provided
Shanorov et al.[13]	278	8.4	Not provided
Jena Biosciences	277	9	Not provided
Sigma-Aldrich	278	8.5	Not provided
Glenn research	277	9	Not provided
Trilink Biotechnologies	279	7.808	Not provided
This study	278	7.948	5.199
Average	278	8.562	5.601^{+}

$^{+}$Average ε_{260} was calculated by multiplying the average ε_{max} by the observed $\varepsilon_{260/278}$ ratio.

Table S6: Molar absorption coefficients of mRNA capping nucleotides as reported in the manufacturers' datasheets.

mRNA cap	ε_{260} ($mM^{-1}cm^{-1}$)
GpppG	24.16
m^7GpppG	22.31
ARCA ($m_2^{7,3'-O}$GpppG)	20.46
$m_3^{2.2.7}$GpppG	21.6
ApppG	27.1
GpppA	27.1
m^7GpppA	25.25
m7Gppp $m^{2'-O}$A	23.43
$m_3^{2.2.7}$GpppA	24.54
ARCA analog ($m_2^{7,3'-O}$Gpppm$^{2'-O}$A)	20.28

Supplemental figures:

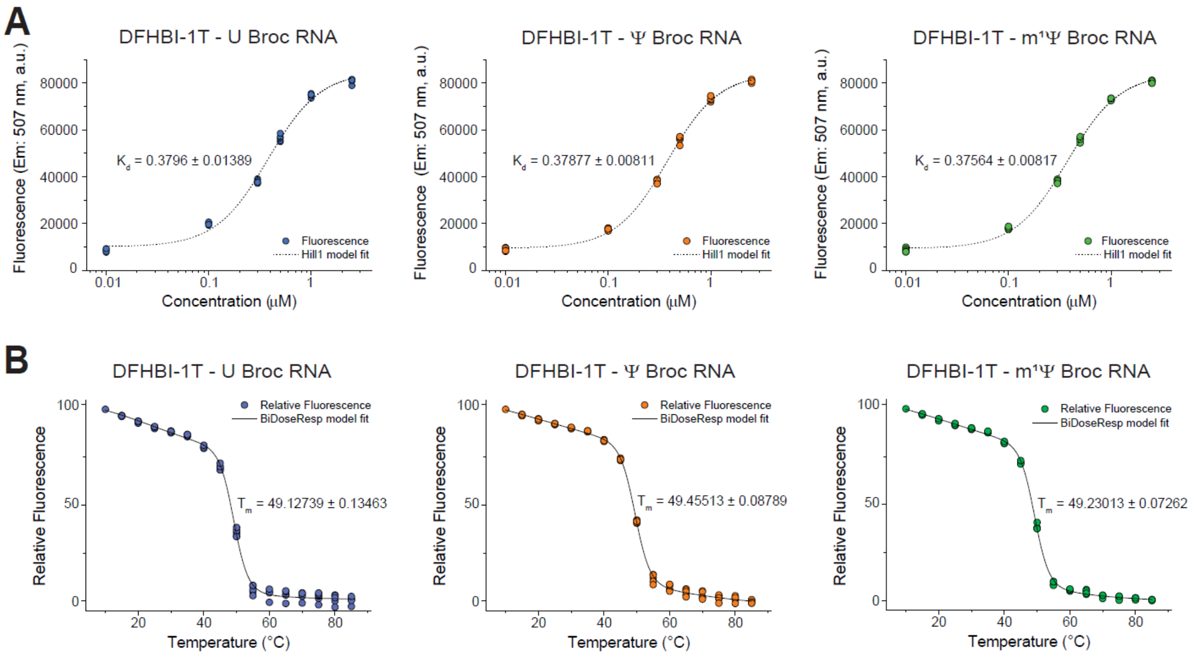

Figure S1: binding and melting curves of mutated Broccoli−DFHBI-1T complexes.

(A) the binding curves of DFHBI-1T onto the U-, Ψ- and m^{1}Ψ-broccoli RNA aptamers are shown. (B) the melting curves of DFHBI-1T onto the U-, Ψ- and m^{1}Ψ-broccoli RNA aptamers are shown. The methods for these experiments are provided in the Supplemental Methods.

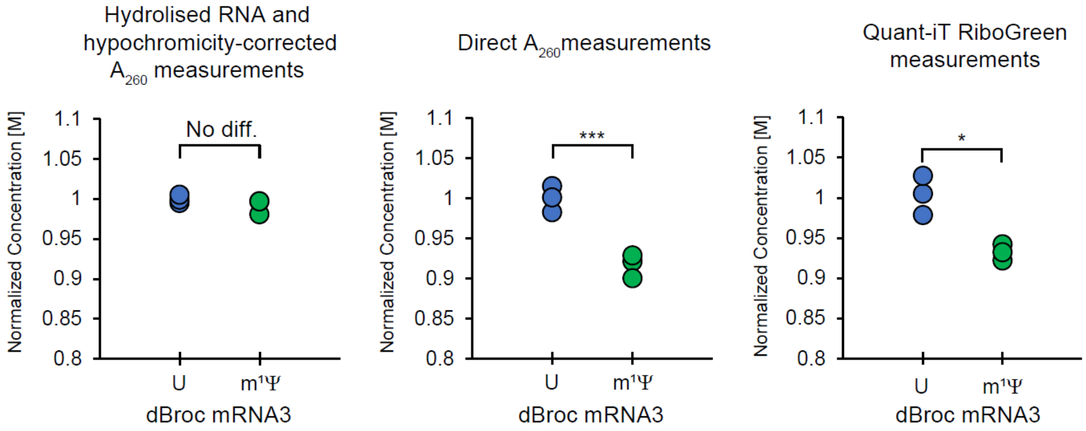

Figure S2: Direct A_{260} measurements and a fluorescence-based assay underestimated modified nucleoside mRNA concentrations. The dBroc-mRNA3 was transcribed using either U or $m^1\Psi$ nucleotides, they were HPLC purified, and their concentration was determined performing RNA hydrolysis (0.8 M NaOH at 37 °C) and implementing a hypochromicity-correction in modified nucleosides. These U- and $m^1\Psi$-mRNAs were then prepared in buffered solution at the same molar concentration, and their concentration was reassessed by performing direct A_{260} measurements, without prior RNA hydrolysis and implementing the extensively used MAC_{260} for ssRNA (40 µg/ml per absorbance unit), or by using a commercially available fluorescence-based assay. We mRNA concentrations measurements are normalized to the mean concentration values in the U-mRNA.

Supplemental Methods:

Determination of photophysical and biochemical properties of mutated Broccoli−DFHBI-1T Complexes

The λ_{max}, relative brightness, dissociation constants and melting points were determined following the methods in reference 15 of the main text.

The emission was measured for solutions using “excess RNA” conditions, to ensure that no free fluorophore contributes to the fluorescence signal. The RNA concentration was 30 µM, while DFHBI-1T concentration was 2 µM. The fluorescence emission was determined in a Fluorolog-3 spectrofluorometer (Horiba Scientific) using the excitation wavelength commonly used for DFHBI-1T, 472 nm, with a side entrance and side exit slits of 3 nm. The integration time was 0.1 seconds, the emission was recorded from 482 nm to 700 nm, with 1 nm increments. The side entrance, front exit and side exit slits were 3 nm.

For the relative brightness, the fluorescence signal of Broccoli-DFHBI-1T complex was compared at different dilutions, using the U-Broccoli-DFHBI-1T as reference.

To calculate dissociation constant (K_D), we titrated increasing concentrations of DFHBI-1T into 50 nM of RNA. The fluorescence at 507 nm wavelength was determined in a Fluorolog-3 spectrofluorometer (Horiba Scientific), using the excitation wavelengths commonly used for DFHBI-1T (Excitation: 472 nm). The resulting data points were fitted to the Hill equation using Origin Pro Software.

To measure the thermostability of RNA-fluorophore complexes, 50 nM of RNA were incubated with 300 µM DFHBI-1T. Then fluorescence values were recorded in 5 °C increments from 10 °C to 85 °C, with a 2-min incubation at each temperature to allow for equilibration, using a CFX96 thermocycler (Bio-rad). The resulting data points were fitted to a biphasic model using Origin Pro Software.

Determination of A260/F507 ratio on dBroc mRNAs:

These methods are described in the Material and Methods section.

References:

1. Cavaluzzi, M.J., and Borer, P.N. (2004). Revised UV extinction coefficients for nucleoside-5′-monophosphates and unpaired DNA and RNA. Nucleic Acids Res *32*, e13. 10.1093/nar/gnh015.

2. Basanta-Sanchez, M., Temple, S., Ansari, S.A., D'Amico, A., and Agris, P.F. (2016). Attomole quantification and global profile of RNA modifications: Epitranscriptome of human neural stem cells. Nucleic Acids Research *44*, e26. 10.1093/nar/gkv971.

3. Yu, C.-T., and Allen, F.W. (1959). Studies of an isomer of uridine isolated from ribonucleic acids. Biochimica et Biophysica Acta *32*, 393–406. 10.1016/0006-3002(59)90612-2.

4. Davis, F.F., and Allen, F.W. (1957). RIBONUCLEIC ACIDS FROM YEAST WHICH CONTAIN A FIFTH NUCLEOTIDE. Journal of Biological Chemistry *227*, 907–915. 10.1016/S0021-9258(18)70770-9.

5. Shapiro, R., and Chambers, R.W. (1961). SYNTHESIS OF PSEUDOURIDINE. J. Am. Chem. Soc. *83*, 3920–3921. 10.1021/ja01479a057.

6. Michelson, A.M., and Cohn, W.E. (1962). Cyclo-pseudouridine and the Configuration of Pseudouridine. Biochemistry *1*, 490–495. 10.1021/bi00909a020.

7. Cohn, W.E. (1960). Pseudouridine, a Carbon-Carbon Linked Ribonucleoside in Ribonucleic Acids: Isolation, Structure, and Chemical Characteristics. Journal of Biological Chemistry *235*, 1488–1498. 10.1016/S0021-9258(18)69432-3.

8. Szer, W. (1965). Secondary structure of poly-5-methylcytidylic acid. Biochemical and Biophysical Research Communications *20*, 182–186. 10.1016/0006-291X(65)90343-8.

9. Martínez-Fernández, L., Pepino, A.J., Segarra-Martí, J., Banyasz, A., Garavelli, M., and Improta, R. (2016). Computing the Absorption and Emission Spectra of 5-Methylcytidine in Different Solvents: A Test-Case for Different Solvation Models. J. Chem. Theory Comput. *12*, 4430–4439. 10.1021/acs.jctc.6b00518.

10. Fox, J.J., Van Praag, D., Wempen, I., Doerr, I.L., Cheong, L., Knoll, J.E., Eidinoff, M.L., Bendich, A., and Brown, G.B. (1959). Thiation of Nucleosides. II. Synthesis of 5-Methyl-2'-deoxycytidine and Related Pyrimidine Nucleosides [1]. J. Am. Chem. Soc. *81*, 178–187. 10.1021/ja01510a042.

11. Ma, C., Cheng, C.C.-W., Chan, C.T.-L., Chan, R.C.-T., and Kwok, W.-M. (2015). Remarkable effects of solvent and substitution on the photo-dynamics of cytosine: a femtosecond broadband time-resolved fluorescence and transient absorption study. Phys. Chem. Chem. Phys. *17*, 19045–19057. 10.1039/C5CP02624E.

12. Shugar, D., and Fox, J.J. (1952). Spectrophotometric studies of nucleic acid derivatives and related compounds as a function of pH: I. Pyrimidines. Biochimica et Biophysica Acta *9*, 199–218. 10.1016/0006-3002(52)90147-9.

13. Sharonov, A., Gustavsson, T., Marguet, S., and Markovitsi, D. (2003). Photophysical properties of 5-methylcytidine. Photochem Photobiol Sci *2*, 362–364. 10.1039/b212664h.
