# Supplementary material for: The mRNACalc webserver accounts for the N1-methylpseudouridine hypochromicity to enable precise nucleoside-modified mRNA quantification
Source: Mol Ther Nucleic Acids. 2024 Mar 11;35(2):102171. doi: 10.1016/j.omtn.2024.102171 (PMC10973171; doi:10.1016/j.omtn.2024.102171)
Supplement: Document S2. Article plus supplemental information [file mmc2.pdf]

Molecular Therapy
Nucleic Acids
Original Article

The mRNACalc webserver accounts for the N1-methylpseudouridine hypochromicity to enable precise nucleoside-modified mRNA quantification

Esteban Finol,[1] Sarah E. Krul,[2,3] Sean J. Hoehn,[2,3] Xudong Lyu,[1] and Carlos E. Crespo-Hernández[2]

[1]Programme in Emerging Infectious Diseases, Duke-NUS Medical School, National University of Singapore, Singapore 169857, Singapore; [2]Department of Chemistry, Case Western Reserve University, Cleveland, OH 44106, USA

Nucleoside-modified messenger RNA (mRNA) technologies necessarily incorporate N1-methylpseudouridine into the mRNA molecules to prevent the over-stimulation of cytoplasmic RNA sensors. Despite this modification, mRNA concentrations remain mostly determined through the measurement of UV absorbance at 260 nm wavelength (A_{260}). Herein, we report that the N1-methylpseudouridine absorbs approximately 40% less UV light at 260 nm than uridine, and its incorporation into mRNAs leads to the under-estimation of nucleoside-modified mRNA concentrations, with 5%–15% error, in an mRNA-sequence-dependent manner. We therefore examined the RNA quantification methods and developed the mRNACalc webserver. It accounts for the molar absorption coefficient of modified nucleotides at 260 nm wavelength, the RNA composition of the mRNA, and the A_{260} of the mRNA sample to enable accurate quantification of nucleoside-modified mRNAs.

INTRODUCTION

The therapeutic use of messenger RNA (mRNA) has sparked great optimism in the development of novel vaccines and therapeutics against a myriad of infectious or as-yet-incurable diseases.[1] The mRNA technology enables the production of antigenic, functional, and/or therapeutic proteins by introducing mRNA into the human body and cells.[2] Since mRNAs act in the cytoplasm transiently, they do not bear any risk of integration into the host cell genome. Most importantly, the mRNA technology enables rapid, cost-efficient, and scalable production, which is free of cellular (cell cultures) or animal materials.[3] Thus, mRNA technologies facilitate manufacturing and allow for a rapid response to emerging infectious diseases, as emphatically underscored by the rapid rollout of COVID-19 mRNA vaccines in many parts of the world. Modified nucleosides, such as pseudouridine (Ψ), N1-methylpseudouridine ($m^1Ψ$), and 5-methylcytidine (m^5C), are often incorporated into the mRNA molecules. Such modifications reduce stimulation of cytoplasmic RNA sensors, such as Toll-like receptors 3 and 7, for improved safety profiles and enhanced mRNA translation.[4,5] However, how modified nucleosides affect mRNA concentration measurements and potentially confound preclinical dosing, efficacy, and toxicology studies, which could make or break further clinical development of any therapeutic, remains undefined.

The determination of RNA concentration often relies on measurements of its UV absorbance at 260 nm wavelength (A_{260}) and the implementation of the Beer-Lambert law.[6] The accuracy of these measurements is scattered by the variable hypochromicity of RNA due to its sequence-dependent folding. The molar absorption coefficient (MAC, or extinction coefficient [ε]) of a folded RNA at 260 nm ($ε_{260}$) is reduced as compared to its unfolded state.[7] This difference is buffer and concentration dependent and arises from changes in the chemical environment of the nucleobases—the main chromophore—due to base pairing, stacking, intermolecular interactions, and other conformational changes. Considering these variabilities, a rough estimation for the MAC_{260} of any single-stranded RNA (ssRNA), 40 μg/mL per absorbance unit, is extensively used, and its associated ±10%–20% error in the estimation of RNA concentration is widely accepted.[6] This error range may suffice to assess dose response for mRNA therapeutics across several orders of magnitude *in cellula* or in *in vivo* experiments, yet it would be valuable to know concentrations at higher accuracy for the development of mRNA technologies. Our particular concern is in measurements of self-amplifying RNAs (saRNAs) and nucleoside-modified mRNAs. The logarithmic amplification of saRNA can convert a 20% accepted error in RNA concentration into several-fold differences in dose response between one experiment and subsequent replicates. The chemical modifications on the nucleobases of mRNA can also induce profound changes in the mRNA MAC hindering the accurate quantification of nucleoside-modified mRNA concentrations.

To attain greater accuracy in RNA quantification, RNA molecules are hydrolyzed prior to UV absorbance determination using a combination of thermal and alkaline hydrolysis.[6,8] The RNA hydrolysis shifts the

Received 14 December 2023; accepted 8 March 2024;
https://doi.org/10.1016/j.omtn.2024.102171.

[3]These authors contributed equally

Correspondence: Esteban Finol, Programme in Emerging Infectious Diseases, Duke-NUS Medical School, National University of Singapore, Singapore 169857, Singapore.
E-mail: esteban.finol@duke-nus.edu.sg
Correspondence: Carlos E. Crespo-Hernández, Department of Chemistry, Case Western Reserve University, Cleveland, OH 44106, USA.
E-mail: carlos.crespo@case.edu

This is an open access article under the CC BY license (http://creativecommons.org/licenses/by/4.0/).

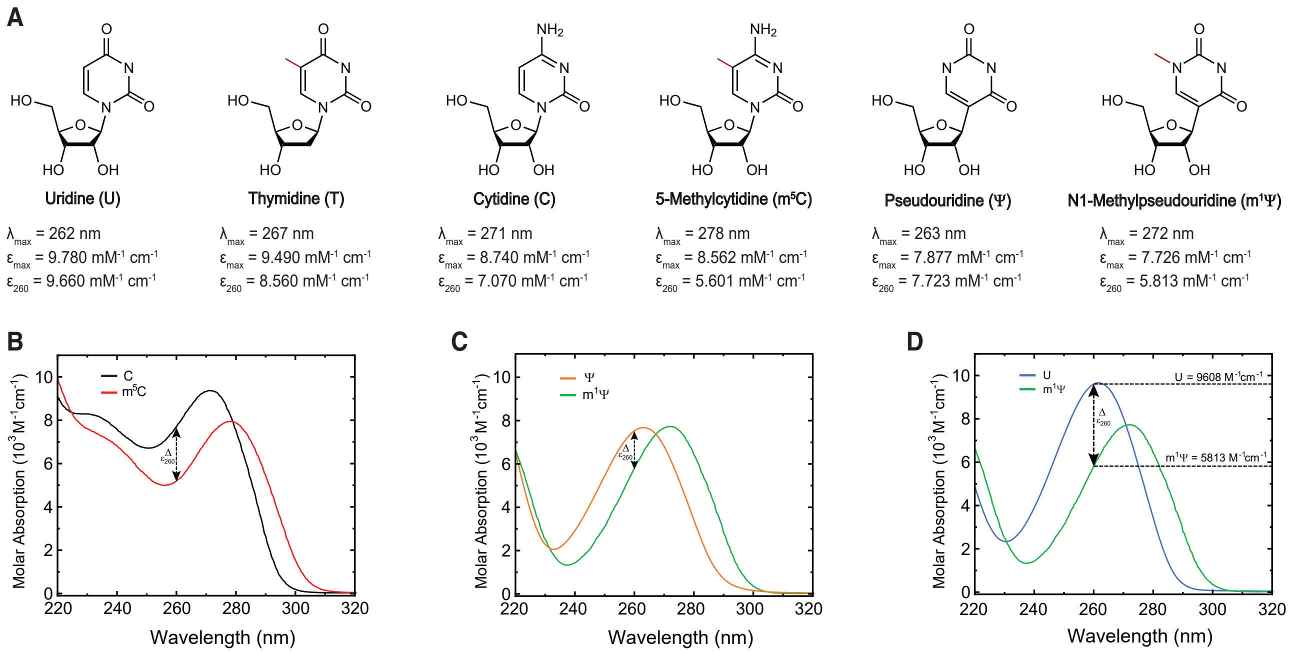

Figure 1. The nucleobase methylation and its bathochromic effect on the UV molar absorption spectra of pyrimidines

(A) Skeletal formula of uridine, thymidine, cytidine, 5-methylcytidine, pseudouridine, and N1-methylpseudouridine. The methyl substituents are highlighted in red. These λ_{max}, ε_{max}, and ε_{260} values are implemented in the mRNACalc webserver. The source of these values is provided in the supplemental information. (B) Steady-state absorption spectra of cytidine (black line) and 5-methylcytidine (red line) at pH 7.4. (C) Steady-state absorption spectra of pseudouridine (orange line) and N1-methylpseudouridine (green line) at pH 7.4. (D) Steady-state absorption spectra of uridine (light blue line) and N1-methylpseudouridine (green line) at pH 7.4. The ε_{260} for U and $m^1\Psi$ are shown.

hypochromic folded state of the RNA to the hyperchromic state of the single monophosphate nucleotides.[9] Since the precise MAC of the four standard nucleotides in aqueous buffered solution is known, the molar absorption of any hydrolyzed mRNA can be calculated as the sum of the molar absorption of its nucleotide compositions. Thus, upon the A_{260} determination, the RNA concentration can be quantified with an error of ∼4% using these methods.[6] The incorporation of modified nucleosides can alter the RNA molar absorption and increase the error of the measurements in an RNA-sequence-dependent manner. Other non-UV-spectroscopic methods relying on the unspecific RNA binding of fluorophores for the determination of RNA concentration may help to overcome any change in the MAC of modified nucleoside mRNA. However, the impact of RNA modifications on the binding affinity of these fluorophores also remains unknown.

Herein, we report our effort to revisit and determine the MAC of modified nucleosides (Ψ, $m^1\Psi$, and m^5C). We also examined three different methods for RNA hydrolysis and provided them along with the mRNACalc webserver. This web tool incorporates the most recently revised MAC_{260} for standard, modified, and mRNA capping nucleosides, allowing the accurate determination of standard and nucleoside-modified mRNAs using UV spectroscopy.

RESULTS

To assess the impact of chemical modifications on the spectrophotometric parameters of pyrimidine nucleosides for mRNA quantification, we determined and compared the molar UV absorption curves of standard nucleosides (U and C) and the modified nucleosides that have recently been employed in nucleoside-modified mRNA technologies (Ψ, $m^1\Psi$, and m^5C). For the cytidine-to-m^5C comparisons, a shift of +7 nm in the peak maximum ($\Delta\lambda_{max}$) was observed with a 20.8% reduction in the ε_{260} for the m^5C nucleoside (Figures 1A and 1B). For the Ψ and $m^1\Psi$ curves, a similar shift was detected ($\Delta\lambda_{max}$ = +9 nm in $m^1\Psi$; Figure 1C), with a reduced molar absorption at 260 nm for $m^1\Psi$ ($\Delta\varepsilon_{260}$ = −22.8%). More importantly, $m^1\Psi$ is hypochromic as compared to uridine at λ_{max} ($\Delta\varepsilon_{max}$ = −21%), and, due to the λ_{max} shift, $m^1\Psi$ absorbs 39.8% less than uridine at 260 nm (Figure 1D), suggesting that $m^1\Psi$-incorporated mRNAs can have reduced MACs.

To assess whether the complete U-to-$m^1\Psi$ substitution alters the UV absorbance of an mRNA, the same mRNA was transcribed using either U, Ψ, or $m^1\Psi$. These mRNAs also encoded a dimeric-Broccoli (dBroc) aptamer in their 3′ untranslated regions (UTRs) (Figure 2A). Once the DFHBI-1T fluorophore was bound to the G-quadruplex in the Broccoli aptamer, the mRNA emitted green light upon excitation.[10] We also confirmed that the brightness, melting point, and affinity of the DFHBI-1T-Broccoli complex are not significantly perturbed by the U-to-Ψ or U-to-$m^1\Psi$ substitution (Table S1; Figure S1). After normalizing the UV absorbance (A_{260}) of each mRNA by its corresponding fluorescence (F_{507}), it was observed that, in practice, the relative UV absorbance of the nucleoside-modified mRNA was

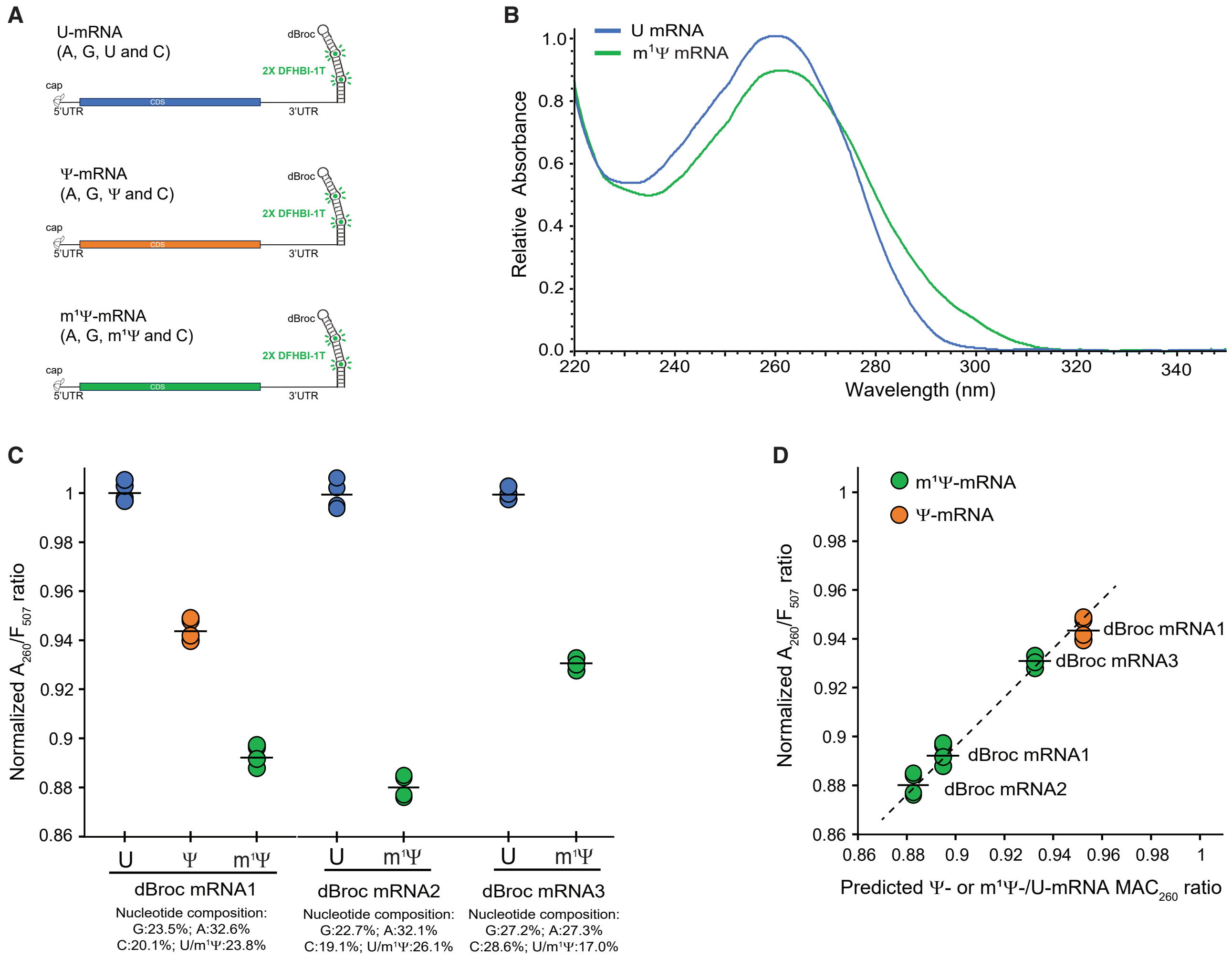

Figure 2. The hypochromicity of nucleoside-modified mRNA can be predicted from their nucleoside composition

(A) Schematic representation of the mRNAs that were designed to determine the normalized A_{260}/F_{507} values. (B) Relative UV absorption curves from mRNAs with uridine or N1-methylpseudouridine nucleosides. They were normalized to the corresponding F_{507} values and plotted relative to the peak maximum of the U-mRNA. (C) The normalized A_{260}/F_{507} values from five replicates of the U-, Ψ-, and m¹Ψ-mRNAs are shown for dBroc-mRNA1. Similar measurements in two additional U- and m¹Ψ-mRNAs are shown. The black lines correspond to the average absorbance. Values are relative to the average absorbance of the U-mRNA. All comparisons of the mean relative A_{260}/F_{507} values were significant (t test; $p < 0.005$). (D) The normalized A_{260}/F_{507} values in (C) were plotted against their predicted hypochromicity using the mRNACalc software.

significantly reduced as compared to the standard mRNA (ΔA_{260} = −10.6%; Figures 2B and 2C). This hypochromicity was also independently observed in two additional mRNAs with either higher or lower $m^1\Psi$ composition (ΔA_{260} = −11.8% and −6.7%, respectively, in Figure 2C). These findings confirmed that $m^1\Psi$-mRNAs are hypochromic and their hypochromicity is dependent on the nucleoside composition. To correct for the observed hypochromicity in nucleoside-modified mRNA, we built the mRNACalc software, which calculates the expected MAC_{260} of a hydrolyzed mRNA. It considers its nucleotide composition and the MAC of standard and modified nucleosides, including the nucleosides in the mRNA cap (documentation in the supplemental information and Tables S2–S6). We used this software to predict MAC_{260} for the different U-, Ψ-, and $m^1\Psi$-dBroc-mRNAs in Figure 2C and plotted their Ψ-/U-mRNA and $m^1\Psi$-/U-mRNA MAC_{260} ratios against the experimentally determined normalized A_{260}/F_{507} ratio (Figure 2D). The observed linearity in this graph corresponds to the expected linearity in the Beer-Lambert law for standard and modified nucleosides and its implementation in ssRNAs, such as mRNA.

To enable accurate measurement of nucleoside-modified mRNA, we also assessed different RNA hydrolysis methods. The modern analytical use of alkaline hydrolysis of RNA has been known since 1922, when Steudel and Peiser demonstrated that 1 M NaOH hydrolyzed yeast RNA whereas thymus DNA resisted the NaOH hydrolysis.[11] The alkali-promoted transesterification of RNA occurs due to the nucleophilic attack of the 2′-OH in the ribose to the 3′,5′-phosphodiester bond, explaining the alkali resistance of the

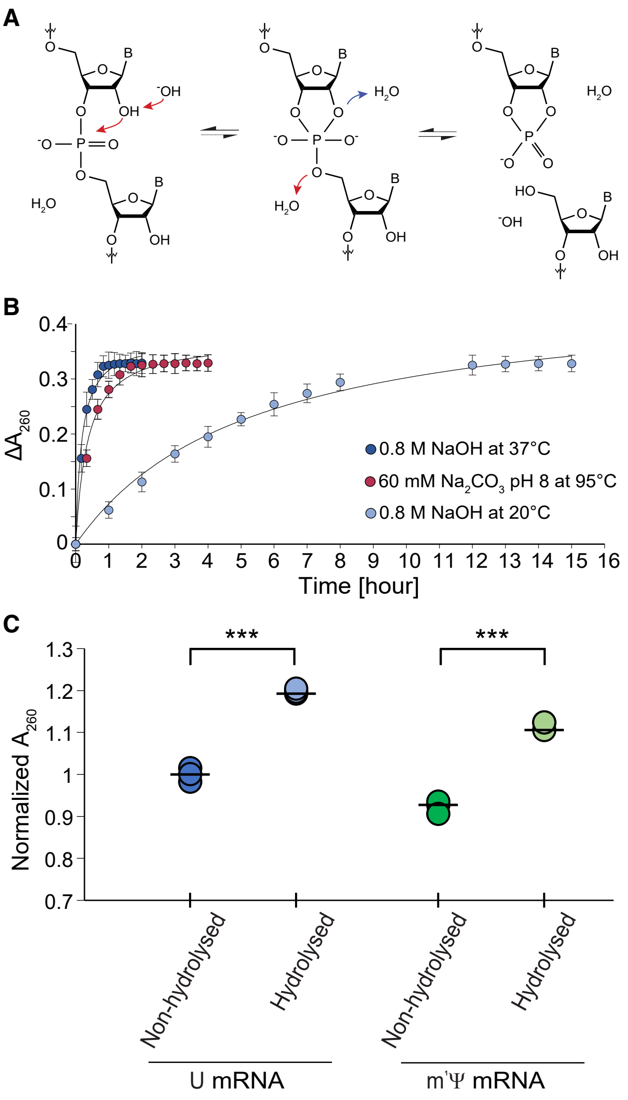

Figure 3. RNA hydrolysis is essential for the determination of mRNA concentrations

(A) Alkali-promoted transesterification allows RNA hydrolysis and mRNA quantification. Under alkaline conditions, the reactive -OH triggers the nucleophilic attack of the 2′-OH on the 3′,5′-phosphodiester linkage, converting the ground-state configuration of RNA into a penta-coordinated intermediate and leading to a 2′3′-cyclic phosphodiester. This cyclic form is then known to form 3′ and 2′ monophosphate nucleotides (data not shown). (B) Thermal and/or alkaline hydrolysis of RNA over time. Yeast RNA was hydrolyzed using three different previously described methods and the ΔA_{260} was determined using a UV spectrophotometer at different intervals. For expedited RNA hydrolysis (1 or 2 h incubation), a combination of thermal and alkaline hydrolyses can be used (dark blue dots, 0.8 M NaOH at 37°C; red dots, 0.5 M Na_2CO_3 [pH 8] at 95°C). For overnight incubation, alkaline hydrolysis suffices (light blue dots, 0.8 M NaOH at 20°C, the last four measurements were performed after an overnight incubation). Dots indicate the mean value of three measurements. Error bars correspond to standard deviations. (C) Hydrolysis of U-mRNA and $m^1\Psi$-mRNA using 0.8 M NaOH at 37°C increases the UV absorption of mRNA. This mRNA corresponds to dBroc-mRNA3 in (C). A_{260} values are normalized to the mean A_{260} values of the non-hydrolyzed U-mRNA.

2′-deoxyribonucleotides (Figure 3A).[12] This reaction is further catalyzed with the introduction of heat. However, the combination of thermal and alkaline hydrolysis, e.g., 1 M NaOH at 95°C, also catalyzes the deamination of cytosine to uridine in a small percentage of residues.[13,14] Thus, a compromise between the two methods is often applied. In our hands, three of such protocols showed a similar increase in A_{260} upon hydrolysis of yeast RNA—a historical standard sample for these methods (Figure 3B). One of these methods (0.8 M NaOH at 37°C) was also applied on U- and $m^1\Psi$-mRNAs (Figure 3C), and the use of RNA hydrolysis indeed increased the A_{260} of both types of mRNA, confirming the importance of performing RNA hydrolysis to remove the effect of RNA folding on the mRNA UV absorption and therefore allow a more accurate determination of mRNA concentrations. We also applied the RNA hydrolysis methods on U- and $m^1\Psi$-mRNAs and determined their concentration by measuring their A260 and using the mRNACalc software to correct for hypochromicity. The concentration of these mRNAs was then reassessed by performing direct A_{260} measurements, without prior RNA hydrolysis and implementing the extensively used MAC_{260} for ssRNA (40 μg/mL per absorbance unit), or by using a commercially available fluorescence-based assay. We could observe that both methods differentially estimated the nucleoside-modified and standard mRNA concentrations, with an underestimation of the $m^1\Psi$-mRNA concentration (Figure S2).

DISCUSSION

Ψ is an isomer of uridine—the standard nucleoside in RNA. Ψ, as opposed to other nucleosides, is a carbon-carbon ribofuranosyl nucleoside, i.e., the uracil nucleobase is linked to the ribose through its fifth carbon instead of an N1 linkage.[15] This unique arrangement places the N1 imino group toward the so-called "C-H" edge of the pyrimidine ring and confers additional properties to this edge in Ψ. This imino hydrogen proton is susceptible to hydrogen bonding, chemical exchange, and chemical modifications such as N1 methylation. Thus, the $m^1\Psi$, as well as the m^5C, represents a modification of the C-H edge of the pyrimidine nucleobase. The influence of a 5-methyl substituent on the UV molar absorption of pyrimidine rings has been well known since the 1940s, when Sister Miriam Michael Stimson showed that a similar 5-methyl modification also differentiates uridine from thymidine and provokes a subtle reduction in molar absorbance (ΔMAC_{max} = −3%) and a shift of the peak maximum ($\Delta\lambda_{max}$ = +5 nm) to a longer wavelength—a bathochromic shift.[16–19] In combination, these two effects provoke a substantial MAC_{260} reduction for the thymidine nucleoside (ΔMAC_{260} = −11.4%). In our study, similar differences were observed for the C-to-m^5C and Ψ-to-$m^1\Psi$ comparisons, with a more pronounced MAC_{260} difference for the U-to-$m^1\Psi$ comparison. Thus, the substitution of uridine by $m^1\Psi$ in mRNA technologies can substantially modify the spectrophotometric properties of the mRNA.

In principle, the modified nucleosides may also promote mRNA folding and reduce its UV absorption. This is particularly relevant for the Ψ modification. Its N1 hydrogen can engage in additional hydrogen bonds, promoting and stabilizing RNA folding. For

instance, the U-to-Ψ substitution in tRNA stabilizes the folded structure that is essential for translation.[20] However, the m^{1}Ψ nucleobase lacks this additional hydrogen bonding capability, and it is expected to have little or no effect on the RNA folding of less structured RNA molecules such as mRNAs. Considering that both Ψ- and m^{1}Ψ-mRNAs followed the anticipated hypochromicity that is associated with the modified nucleosides' hypochromicity at 260 nm wavelength (Figure 1) and their abundance in the mRNA (Figure 2C), rather than the expected distinct contribution of Ψ and m^{1}Ψ to RNA folding, we can conclude that the observed reduction in the UV absorption of nucleoside-modified mRNA is mainly determined by the nucleobase composition and the intrinsic MAC of the nucleosides in the purified mRNAs. Importantly, the UV absorption spectrum of the m^{1}Ψ-mRNA also depicted a broad absorption peak and a bathochromic shift, which brings about additional implications for the assessment of the RNA sample purity (Figure 2B; supplemental information). These findings indicate that for accurate determination of nucleoside-modified mRNA concentrations and proper interpretation of dose-ranging preclinical studies, the reported UV spectroscopic differences must be accounted for. Otherwise, nucleoside-modified mRNA concentrations may be underestimated by 5%–15% depending on the proportion of m^{1}Ψ in the mRNA composition.

Considering that traditional methods underestimate the nucleoside-modified mRNA concentrations and to ease the implementation of the reported UV absorption parameters, we provide the mRNACalc software as an open-source webserver to calculate the MAC_{260} for nucleoside-modified mRNAs. It accounts for the hypochromicity of modified nucleosides as well as for the nucleoside composition of the mRNA, including the mRNA cap. Once the RNA sequence, the A_{260}, and the RNA stock volume values are provided as input, the mRNACalc webserver calculates the RNA stock concentration in nM and ng/μL and the total RNA mass in μmole and μg. The webserver also includes the revisited experimental protocols and a workflow that implements a linear regression model from multiple measurements at serial dilutions (Figure 4). This workflow aims at reducing the impact of sample handling variation. Hence, the mRNACalc webserver represents a freely available and all-inclusive tool for the determination of nucleoside-modified mRNA concentrations using UV spectroscopy.

MATERIALS AND METHODS

The Beer-Lambert experiments

Ψ (≥98% purity), m^{5}C (≥99% purity), cytidine (99% purity), and uridine (99% purity) were purchased from Sigma-Aldrich. m^{1}Ψ (>95% purity) was purchased from Biosynth Carbosynth. They were used as received. Phosphate buffer solutions with a total phosphate concentration of 16 mM from monosodium and disodium phosphate salts dissociated in ultrapure water (Millipore) were freshly prepared on the day of each experiment. The pH of the solution was adjusted using 0.1 M solutions of NaOH and HCl to the desired pH of 7.4 (±0.1 pH units). Steady-state absorption was recorded using a Cary 100 spectrometer. Serial dilutions of known concentration were carried out such that the absorbance reading at the respective lambda maximum (local maximum absorbance) remained below 1.0, within the linear range of the instrument. The MACs were experimentally determined using the slope from the linear regression from plotting absorbance versus concentration. The correlation constant for the linear regression analysis of the Beer-Lambert's law data for determining molar absorption constants was >0.9999, showing a strong linear relationship.

mRNA *in vitro* transcription and purification

The plasmid DNA template (pUCIDT plasmid) was grown in DH5 alpha *E. coli* (New England Biolabs) in 300 mL Luria-Bertani broth supplemented with kanamycin (50 μg/mL), and a maxi preparation was performed using the QIAGEN Plasmid Plus Maxi Kit following manufacturer instructions. The plasmid encoded a T7 promoter followed by the mCherry gene with a degradation tag (1,449 nucleotides) plus the 3′ and 5′ UTRs of the BNT162b2 mRNA vaccine (541 nucleotides). The double broccoli aptamer was encoded within the poly-adenine region in the 3′ UTR. The plasmid was linearized by EcoRV restriction enzyme digestion at the end of the 3′ UTR.

A standard T7 transcription reaction included 30 mM Tris-HCl (pH 7.9), 2 mM spermidine, 30 mM $MgCl_2$, 5 mM NaCl, 10 mM DTT, 50 μg/mL BSA (New England Biolabs), 0.005% Triton X-100, 2% polyethylene glycol (PEG8000), 5 mM of each triphosphate ribonucleotide (standard nucleotides were purchased from Jena Bioscience GmbH and Ψ and m^{1}Ψ from BOC Sciences), 2 μM linearized plasmid DNA template, 3.5 μM T7 RNA polymerase (in-house produced and purified), and 0.0025 units of *E. coli* inorganic PPase (New England Biolabs). All reagents were purchased from Sigma-Aldrich unless otherwise stated. The reactions were incubated at 37°C for 2.5 h and stopped by the addition of 500 mM EDTA (pH 8) to a final concentration of 35 mM.

The mRNA was purified using anion-exchange chromatography. A PRP-X600 anion-exchange column (Hamilton Company) was equilibrated in buffer A (85:15 100 mM Tris [pH 8]/acetonitrile). RNA samples were loaded onto the column at a flow rate of 3 mL/min and eluted with a 40 min gradient of 0%–40% buffer B (85:15 100 mM Tris, 2.5 M LiCl [pH 8]/acetonitrile). Fractions containing the mRNA were collected, and the mRNA molecules were precipitated using standard butanol extraction.[21] The purity of the mRNA preparation was assessed using high-resolution automated electrophoresis in the Agilent 2100 Bioanalyzer system using the Bioanalyzer RNA 6000 pico assay (Agilent Technologies).

Determination of the mRNA UV absorption spectrum

To determine the UV absorption spectrum of mRNAs, the mRNA stocks were diluted to approximately 25 nM into a buffer containing 40 mM HEPES (pH 7.4), 5 mM $MgCl_2$, and 100 mM KCl to a final volume of 2 mL. Five independent mRNA samples were prepared per mRNA set (U-, Ψ-, and m^{1}Ψ-mRNAs). The UV absorption spectra were recorded for each mRNA sample using a UV-3600i plus UV-visible (UV-vis) spectrophotometer (Shimadzu Corp.).

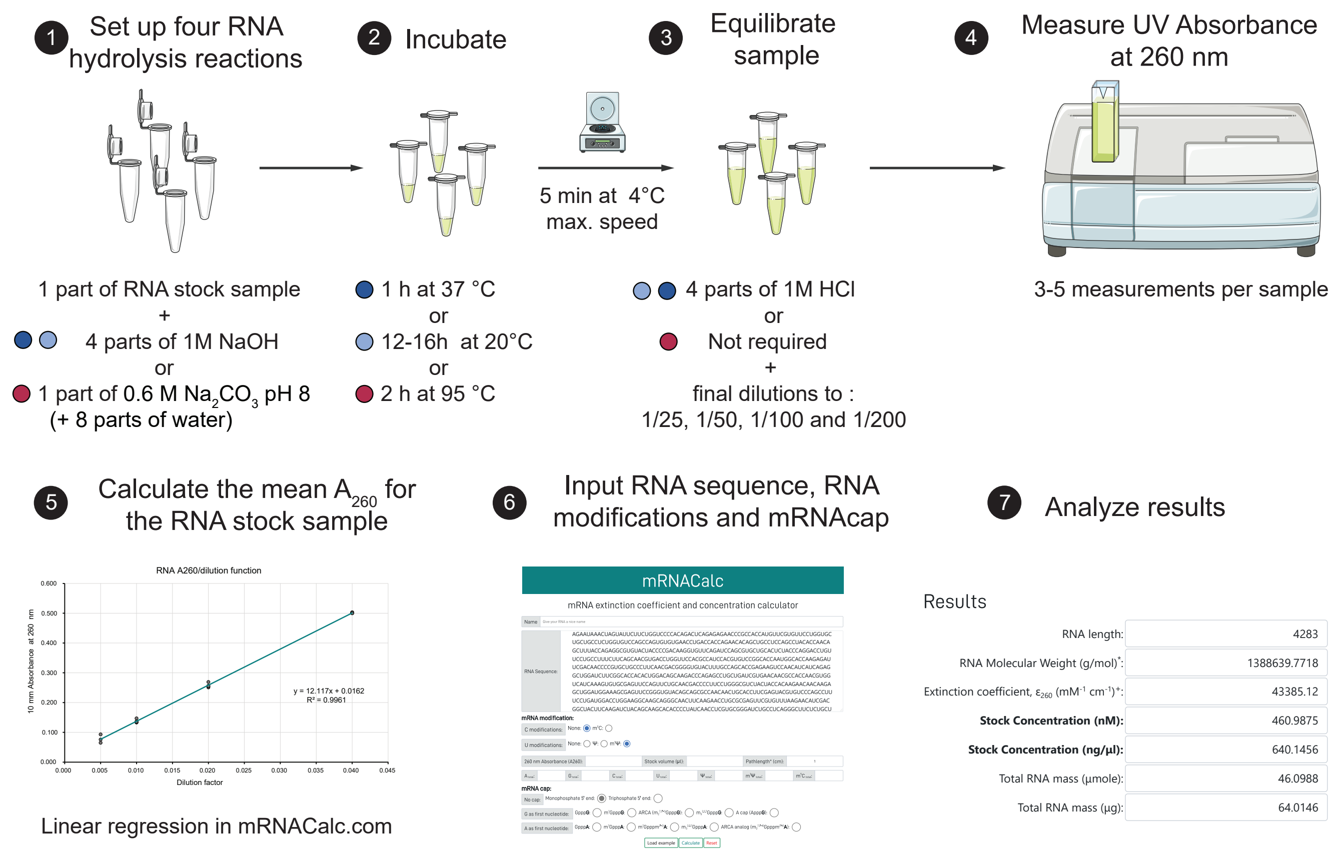

Figure 4. Experimental workflow for the determination of RNA concentration using the mRNACalc webserver
The colored dots refer to the different RNA hydrolysis methods in Figure 3B.

Excitation-emission experiments on the DFHBI-1T-bound mRNAs

After UV absorption determination, the mRNA samples were bound to the DFHBI-1T fluorophore by adding 100 μM DFHBI-1T and 100% DMSO to a 500 nM concentration into the 2 mL mRNA samples. Fluorescence was measured with a Fluorolog-3 spectrofluorometer (Horiba Scientific) using the excitation and emission wavelengths commonly used for DFHBI-1T (excitation: 472 nm, emission: 507 nm).[10]

Determination of the relative UV absorbance (A_{260})

The A_{260}/F_{507} ratios were calculated for each mRNA sample. The mean A_{260}/F_{507} values for U-, Ψ-, and m^{1}Ψ-mRNAs were calculated. The A_{260}/F_{507} values of each sample were normalized using the mean A_{260}/F_{507} value from the U-mRNA as reference, and they were plotted in a dot plot. The t tests were applied to compare the mean A_{260}/F_{507} values across each pair of mRNA sets using a p value of 0.005 as the cut-off of significance.

Methods of RNA hydrolysis

Two methods of RNA hydrolysis were tested in this study. Torula yeast RNA was used as standard RNA sample (Sigma-Aldrich). The yeast RNA stock was prepared at 1,000 μg/μL in water. Thus, after 1/25 dilution, the UV absorbance of this RNA sample would be within the linear range of the instrument (UV-3600i plus UV-vis spectrophotometer, Shimadzu Corp.).

The most extensively used alkaline RNA hydrolysis method involves adding 1 part RNA and 4 parts 1 M NaOH and incubating them at 37°C for 1 h.[22] To test this method, twelve yeast RNA samples were hydrolyzed. Every 10 min, a sample was neutralized with 4 parts 1 M HCl and diluted to 1/25 with 16 parts water. Three UV absorbance measurements were performed on every sample. Similarly, a room temperature variation of this method is often used for overnight RNA hydrolysis. Therefore, twelve RNA samples were hydrolyzed and incubated at 20°C for up to 15 h. Samples were neutralized and diluted hourly followed by three UV absorbance measurements.

A second method of thermal hydrolysis at neutral pH was also tested.[8] To test this method, twelve yeast RNA samples hydrolyzed (1 part RNA in 9 parts 60 mM Na_2CO_3 [pH 8]) with an incubation of at 95°C for up to 2 h. Every 20 min, a sample was diluted to 1/25 with 15 parts water, and three UV absorption measurements were performed on every sample.

DATA AND CODE AVAILABILITY

The data that support the findings of this study are available from the corresponding author upon reasonable request. The webserver is available at https://www.mrnacalc.com. The website is free and open to all users, and there is no login requirement. The HTML script for the mRNACalc webserver is available under a GNU general public license from https://github.com/estebanfbfc/mRNACalc. It can be downloaded free of charge and run locally without internet access.

SUPPLEMENTAL INFORMATION

Supplemental information can be found online at https://doi.org/10.1016/j.omtn.2024.102171.

ACKNOWLEDGMENTS

The authors would also like to thank Prof. Eng Eong Ooi for his invaluable advice and generosity throughout this work and Prof. Guillermo C. Bazan for providing access to the UV-vis and fluorescence spectrometers in his laboratory. This work was supported by the National Medical Research Council of Singapore through an Open Fund - Large Collaborative Grant, granted to Prof. Eng Eong Ooi, and by the National Science Foundation (grant no. CHE-2246805), granted to C.E.C-H.

AUTHOR CONTRIBUTIONS

E.F. conceived the study. E.F. and C.E.C.-H. supervised the project. E.F. developed the mRNACalc webserver. S.E.K. and S.J.H. performed the Beer-Lambert experiments and prepared the corresponding figure panel. E.F. and X.L. performed the relative absorbance of mRNA experiments and analyzed the data. E.F. prepared figures, wrote the initial draft of the manuscript, and edited the submitted version of the manuscript with contributions from all the authors.

DECLARATION OF INTERESTS

The authors declare no competing interests.

REFERENCES

1. Chaudhary, N., Weissman, D., and Whitehead, K.A. (2021). mRNA vaccines for infectious diseases: principles, delivery and clinical translation. Nat. Rev. Drug Discov. *20*, 817–838. https://doi.org/10.1038/s41573-021-00283-5.
2. Sahin, U., Karikó, K., and Türeci, Ö. (2014). mRNA-based therapeutics — developing a new class of drugs. Nat. Rev. Drug Discov. *13*, 759–780. https://doi.org/10.1038/nrd4278.
3. Webb, C., Ip, S., Bathula, N.V., Popova, P., Soriano, S.K.V., Ly, H.H., Eryilmaz, B., Nguyen Huu, V.A., Broadhead, R., Rabel, M., et al. (2022). Current Status and Future Perspectives on MRNA Drug Manufacturing. Mol. Pharm. *19*, 1047–1058. https://doi.org/10.1021/acs.molpharmaceut.2c00010.
4. Karikó, K., Muramatsu, H., Welsh, F.A., Ludwig, J., Kato, H., Akira, S., and Weissman, D. (2008). Incorporation of Pseudouridine Into mRNA Yields Superior Nonimmunogenic Vector With Increased Translational Capacity and Biological Stability. Mol. Ther. *16*, 1833–1840. https://doi.org/10.1038/mt.2008.200.
5. Andries, O., Mc Cafferty, S., De Smedt, S.C., Weiss, R., Sanders, N.N., and Kitada, T. (2015). N1-methylpseudouridine-incorporated mRNA outperforms pseudouridine-incorporated mRNA by providing enhanced protein expression and reduced immunogenicity in mammalian cell lines and mice. J. Control. Release *217*, 337–344. https://doi.org/10.1016/j.jconrel.2015.08.051.
6. Cavaluzzi, M.J., and Borer, P.N. (2004). Revised UV extinction coefficients for nucleoside-5′-monophosphates and unpaired DNA and RNA. Nucleic Acids Res. *32*, e13. https://doi.org/10.1093/nar/gnh015.
7. Tinoco, I., Jr. (1960). Hypochromism in Polynucleotides. J. Am. Chem. Soc. *82*, 4785–4790. https://doi.org/10.1021/ja01503a007.
8. Wilson, S.C., Cohen, D.T., Wang, X.C., and Hammond, M.C. (2014). A neutral pH thermal hydrolysis method for quantification of structured RNAs. RNA *20*, 1153–1160. https://doi.org/10.1261/rna.045856.114.
9. Doty, P., Boedtker, H., Fresco, J.R., Haselkorn, R., and Litt, M. (1959). Secondary structure in ribonucleic acids. Proc. Natl. Acad. Sci. USA *45*, 482–499. https://doi.org/10.1073/pnas.45.4.482.
10. Filonov, G.S., Moon, J.D., Svensen, N., and Jaffrey, S.R. (2014). Broccoli: Rapid Selection of an RNA Mimic of Green Fluorescent Protein by Fluorescence-Based Selection and Directed Evolution. J. Am. Chem. Soc. *136*, 16299–16308. https://doi.org/10.1021/ja508478x.
11. Steudel, H., and Peiser, E. (1922). Über Nucleinsäure-Eiweißverbindungen. Bio. Chem. *122*, 298–306. https://doi.org/10.1515/bchm2.1922.122.4-6.298.
12. Lipkin, D., Talbert, P.T., and Cohn, M. (1954). The Mechanism of the Alkaline Hydrolysis of Ribonucleic Acids. J. Am. Chem. Soc. *76*, 2871–2872. https://doi.org/10.1021/ja01640a004.
13. Wang, R.Y., Kuo, K.C., Gehrke, C.W., Huang, L.-H., and Ehrlich, M. (1982). Heat- and alkali-induced deamination of 5-methylcytosine and cytosine residues in DNA. Biochim. Biophys. Acta *697*, 371–377. https://doi.org/10.1016/0167-4781(82)90101-4.
14. Shen, J.C., Rideout, W.M., and Jones, P.A. (1994). The rate of hydrolytic deamination of 5-methylcytosine in double-stranded DNA. Nucleic Acids Res. *22*, 972–976. https://doi.org/10.1093/nar/22.6.972.
15. Cohn, W.E. (1960). Pseudouridine, a Carbon-Carbon Linked Ribonucleoside in Ribonucleic Acids: Isolation, Structure, and Chemical Characteristics. J. Biol. Chem. *235*, 1488–1498. https://doi.org/10.1016/S0021-9258(18)69432-3.
16. Stimson, M.M. (1949). The Ultraviolet Absorption Spectra of Some Pyrimidines. Chemical Structure and the Effect of pH on the Position of λmax. J. Am. Chem. Soc. *71*, 1470–1474. https://doi.org/10.1021/ja01172a093.
17. Sharonov, A., Gustavsson, T., Marguet, S., and Markovitsi, D. (2003). Photophysical properties of 5-methylcytidine. Photochem. Photobiol. Sci. *2*, 362–364. https://doi.org/10.1039/b212664h.
18. Shugar, D., and Fox, J.J. (1952). Spectrophotometric studies of nucleic acid derivatives and related compounds as a function of pH: I. Pyrimidines. Biochim. Biophys. Acta *9*, 199–218. https://doi.org/10.1016/0006-3002(52)90147-9.
19. Rabczenko, A., and Shugar, D. (1971). Studies on the conformation of nucleosides, dinucleoside monophosphates and homopolynucleotides containing uracil or thymine base residues, and ribose, deoxyribose or 2'-O-methylribose. Acta Biochim. Pol. *18*, 387–402.
20. Lorenz, C., Lünse, C.E., and Mörl, M. (2017). tRNA Modifications: Impact on Structure and Thermal Adaptation. Biomolecules *7*, 35. https://doi.org/10.3390/biom7020035.
21. Green, M.R., and Sambrook, J. (2017). Concentrating Nucleic Acids by Extraction with Butanol. Cold Spring Harb. Protoc. *2017*. pdb.prot093401. https://doi.org/10.1101/pdb.prot093401.
22. Bock, R.M. (1967). [29] Alkaline hydrolysis of RNA. In Methods in Enzymology Nucleic Acids, Part A. (Academic Press), pp. 224–228. https://doi.org/10.1016/S0076-6879(67)12035-1.

OMTN, Volume *35*

Supplemental information

The mRNACalc webserver accounts for the N1-methylpseudouridine hypochromicity to enable precise nucleoside-modified mRNA quantification

Esteban Finol, Sarah E. Krul, Sean J. Hoehn, Xudong Lyu, and Carlos E. Crespo-Hernández

Supplemental Material

On the calculations and parameters in the mRNACalc webserver

- The mRNA molar absorption coefficient (ε) is calculated from the sum of the individual nucleotide extinction coefficients as determined by:

$$\varepsilon_{mRNA} = n_A\varepsilon_A + n_G\varepsilon_G + n_C\varepsilon_C \; or \; n_{m^5C}\varepsilon_{m^5C} + n_U\varepsilon_U \; or \; n_{\Psi}\varepsilon_{\Psi} \; or \; n_{m^1\Psi}\varepsilon_{m^1\Psi} + \varepsilon_{cap}$$

  Where n_N corresponds to the number of each type of nucleotide, N, in the mRNA and ε_N to the molar absorption coefficient for each type of nucleotide, including the capping nucleotide (ε_{cap}).
- The mRNA molecular weight is calculated as the sum of the nucleotide composition mass as RNA-incorporated monophosphate nucleotides.

$$MW_{mRNA} = n_AMW_A + n_GMW_G + n_CMW_C \; or \; n_{m^5C}MW_{m^5C} + n_UMW_U \; or \; n_{\Psi}MW_{\Psi} \; or \; n_{m^1\Psi}MW_{m^1\Psi} + MW_{cap}$$

- The mRNA molar concentration is calculated using the Beer-Lambert equation:

$$Concentration\ (M) = \frac{A260}{\varepsilon_{mRNA} * cm^{-1}}$$

  The mRNA molar concentration is presented in the nM and ng/µl scales in the webserver.

On the molar absorption coefficients of nucleosides/nucleotides

For standard nucleotides, the mRNACalc webserver implements the ε_{260} parameters in Table S2. The parameters from Cavaluzzi et al. were obtained after accurate measurements of nucleotides concentration using nuclear magnetic resonance spectroscopy.

For the modified nucleosides/nucleotides, we have determined and searched for ε_{max} and ε_{260} parameters in the literature and in the datasheet of Ψ, $m^1\Psi$, and m^5C manufacturers, which are summarized in Tables S3, S4 and S5.

Considering the extensive variability across the published and manufacturer-provided values for m^5C and Ψ, the mRNACalc webserver implements the average ε_{260} values. For $m^1\Psi$, the mRNACalc webserver implements the ε_{260} value that was obtained for this study, due to the limited number of previously reported values.

For the mRNA capping nucleotides, the mRNACalc webserver implements the ε_{260} values provided by the manufactures (Table S6). In few cases, the ε_{260} values were not available, the independent ε_{260} values of the two nucleotides were summed up.

Overall, these mRNA cap parameters were only implemented for completeness, and they can be considered as rough estimations, despite their contribution to an mRNA UV absorption is rather negligible.

Important note: The molar absorption parameters, herein compiled, correspond to either nucleosides or nucleotides in aqueous buffered solution (pH 7 – 8). Considering that the contribution of the phosphate group to the molar absorption of nucleotides is negligible, they have been considered for their implementation in the mRNACalc webserver indiscriminately.

On the purity of RNA samples:

The presence of impurities in nucleic acid samples is often assessed using the $A_{260:280}$ and $A_{260:230}$ ratios. For pure RNA the $A_{260:280}$ ratio is ~ 2.0. This ratio is commonly used to assess the amount of protein contamination, since proteins absorb at 280 nm. Similarly, the $A_{260:230}$ ratio for pure RNA is often slightly higher than the $A_{260:280}$ ratio, ranging from 2.0 to 2.2. Residual chemical contamination (phenol, butanol, carbohydrates, guanidine, and others) from the RNA purification method can increase the A_{230} and reduce the $A_{260:230}$ ratio.

From our experience, the assessment of purity for the $m^1\Psi$ modified mRNA samples requires shifting the wavelength for these ratios to $A_{264:284}$ and $A_{264:234}$ due to the bathochromic shift in the mRNA absorption curve (Figure S3). Which leads to:

- a reduced A_{260} due to the λ_{max} shift (λ_{max} at ~264 nm),
- an increased A_{230} due to the shift on the curve trough (λ_{min}) to ~234 nm,
- and an increased A_{280} due to the absorbance peak shift and broadening. The broadening arises due to the wider range of λ_{max} values in the RNA composition (standard mRNA= 252 to 263 nm, $m^1\Psi$ modified mRNA= 252 to 272 nm) as well as, due to the broader absorption peak of $m^1\Psi$ (as determined by the peak width at the trough level: Urd= 52.5 nm vs $m^1\Psi$= 57.0 nm).

Thus, $A_{264:284}$ and $A_{264:234}$ ratios should be interpreted in the same manner as the $A_{260:280}$ and $A_{260:230}$ ratios, respectively. Alternatively, the $A_{260:280}$ ratio can be accepted at 1.9 and the $A_{260:220}$ ratio can range between 1.9 to 2.1.

Importantly, m^5C-modified mRNA should show similar modifications in the UV molar absorption spectrum and the proposed shifted ratios may be applied as well.

Supplemental tables:

Table S1: photophysical and biochemical properties of mutated Broccoli−DFHBI-1T complexes.

Complex	λ_{max} (nm)	Relative brightness*	K_D (nM)$^+$	T_m (°C)$^+$
U-Broc−DFHBI-1T	507	------	360	48
U-Broc−DFHBI-1T	507	1.000 ± 0.002	379.6 ± 13.89	49.13 ± 0.13
Ψ-Broc-DFHBI-1T	507	1.005 ± 0.004	378.7 ± 8.11	49.46 ± 0.09
m^{1}Ψ-Broc-DFHBI-1T	507	1.004 ± 0.003	375.6 ± 8.17	49.23 ± 0.07

*Relative to the U-Broc-DFHBI-1T complex. Data are shown as mean ± SD.
$^+$ Data are shown as fitted K_D ± Error of the fit or fitted T_m ± Error of the fit.

Table S2: Molar absorption coefficients of standard nucleosides as reported in Cavaluzzi et al.

Standard nucleosides	λ_{max} (nm)	ε_{max} (mM^{-1}cm^{-1})	ε_{260} (mM^{-1}cm^{-1})
Uridine	262	9.78	9.66
Uridine*	262	9.66	9.60
Thymidine	267	9.49	8.56
Cytidine	271	8.74	7.07
Cytidine*	271	9.34	7.67
Guanosine	252	14.09	12.08
Adenosine	259	15.04	15.02

Source: Cavaluzzi et al. [1] and *this study.

Table S3: Molar absorption coefficients of pseudouridine as reported in the literature and manufacturers' datasheets.

Source	λ_{max} (nm)	ε_{max} (mM^{-1}cm^{-1})	ε_{260} (mM^{-1}cm^{-1})
Basanta-Sanchez et al. [2]	262	7.583	7.492
Yu & Allen.[3]	263	7.5	Not provided
David & Allen. [4]	263	8.4	8.3
Shapiro & Chambers. [5]	262	7.9	Not provided
Michelson & Cohn. [6]	262	8.0	Not provided
Cohn. [7]	263	8.1	Not provided
Jena Biosciences	265	7.9	Not provided
Trilink Biotechnologies	262	7.546	Not provided
This study	263	7.677	7.527
Average	263	7.877	7.723$^+$

$^+$Average ε_{260} was calculated by multiplying the average ε_{max} by the observed $\varepsilon_{260/263}$ ratio.

Table S4: Molar absorption coefficients of N1-methylpseudouridine as reported in the literature and manufacturers' datasheets.

Source	λ_{max} (nm)	ε_{max} ($mM^{-1}cm^{-1}$)	ε_{260} ($mM^{-1}cm^{-1}$)
Roche	271	7.3	Not provided
Trilink Biotechnologies	271	8.877	Not provided
This study	272	7.726	5.813
Average	271	7.967	5.994^{+}

$^{+}$Average ε_{260} was calculated by multiplying the average ε_{max} by the observed $\varepsilon_{260/272}$ ratio.

Table S5: Molar absorption coefficients of 5-methylcytidine as reported in the literature and manufacturers' datasheets.

Source	λ_{max} (nm)	ε_{max} ($mM^{-1}cm^{-1}$)	ε_{260} ($mM^{-1}cm^{-1}$)
Szer. [8]	278.5	8.8	Not provided
Martínez-Fernández et al.[9]	278	8.92	Not provided
Fox et al.[10]	277.5	8.88	Not provided
Ma et al.[11]	278	8.871	Not provided
Fox & Shugar. [12]	276	8.05	Not provided
Shanorov et al.[13]	278	8.4	Not provided
Jena Biosciences	277	9	Not provided
Sigma-Aldrich	278	8.5	Not provided
Glenn research	277	9	Not provided
Trilink Biotechnologies	279	7.808	Not provided
This study	278	7.948	5.199
Average	278	8.562	5.601^{+}

$^{+}$Average ε_{260} was calculated by multiplying the average ε_{max} by the observed $\varepsilon_{260/278}$ ratio.

Table S6: Molar absorption coefficients of mRNA capping nucleotides as reported in the manufacturers' datasheets.

mRNA cap	ε_{260} ($mM^{-1}cm^{-1}$)
GpppG	24.16
m^7GpppG	22.31
ARCA ($m_2^{7,3'\text{-}O}$GpppG)	20.46
$m_3^{2.2.7}$GpppG	21.6
ApppG	27.1
GpppA	27.1
m^7GpppA	25.25
m7Gppp $m^{2'\text{-}O}$A	23.43
$m_3^{2.2.7}$GpppA	24.54
ARCA analog ($m_2^{7,3'\text{-}O}$Gpppm$^{2'\text{-}O}$A)	20.28

Supplemental figures:

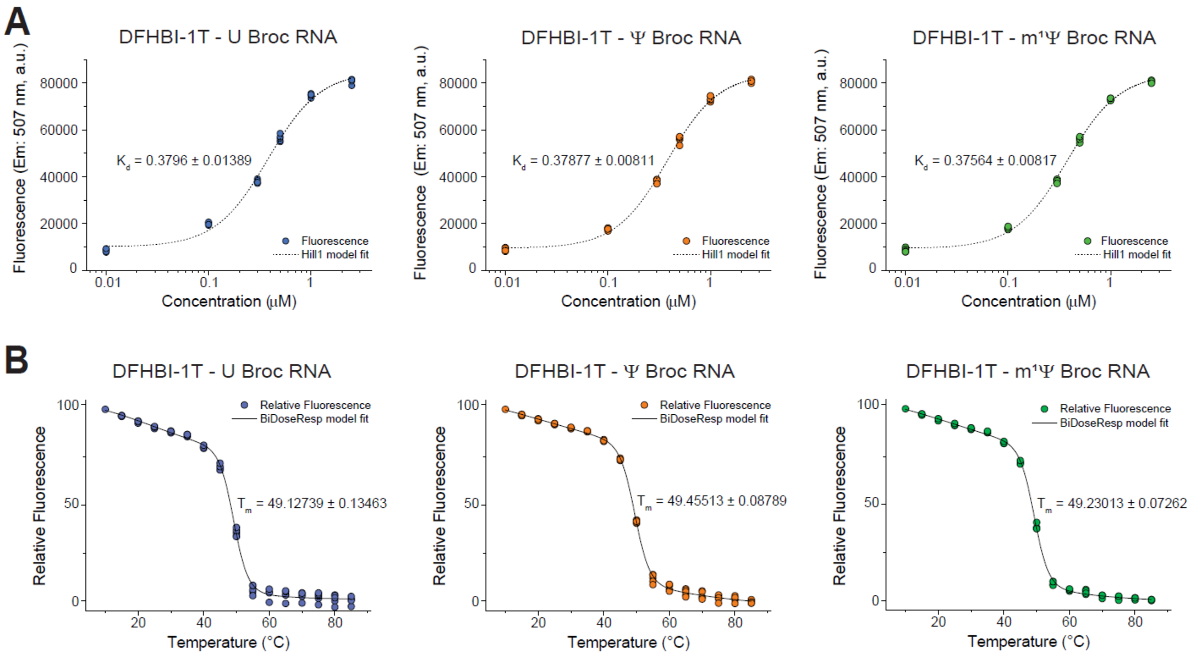

Figure S1: binding and melting curves of mutated Broccoli−DFHBI-1T complexes.

(A) the binding curves of DFHBI-1T onto the U-, Ψ- and m^{1}Ψ-broccoli RNA aptamers are shown. (B) the melting curves of DFHBI-1T onto the U-, Ψ- and m^{1}Ψ-broccoli RNA aptamers are shown. The methods for these experiments are provided in the Supplemental Methods.

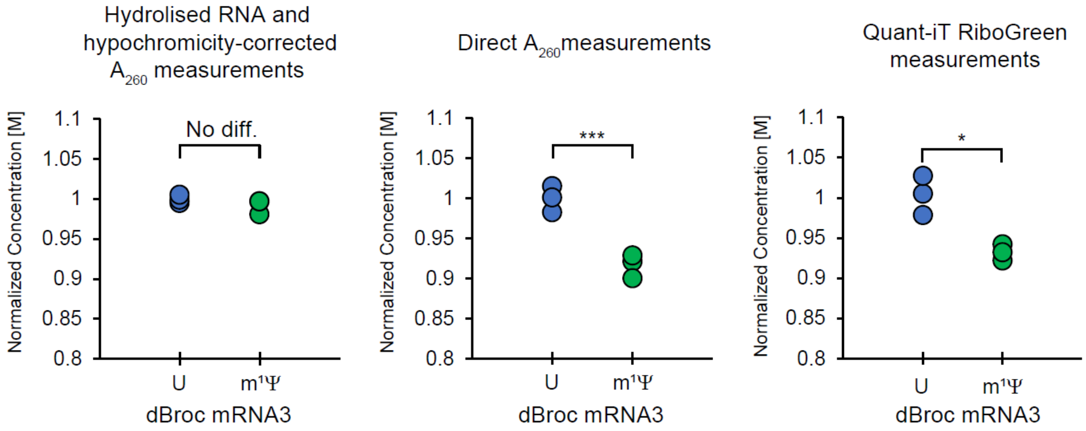

Figure S2: Direct A_{260} measurements and a fluorescence-based assay underestimated modified nucleoside mRNA concentrations. The dBroc-mRNA3 was transcribed using either U or $m^1\Psi$ nucleotides, they were HPLC purified, and their concentration was determined performing RNA hydrolysis (0.8 M NaOH at 37 °C) and implementing a hypochromicity-correction in modified nucleosides. These U- and $m^1\Psi$-mRNAs were then prepared in buffered solution at the same molar concentration, and their concentration was reassessed by performing direct A_{260} measurements, without prior RNA hydrolysis and implementing the extensively used MAC_{260} for ssRNA (40 µg/ml per absorbance unit), or by using a commercially available fluorescence-based assay. We mRNA concentrations measurements are normalized to the mean concentration values in the U-mRNA.

Supplemental Methods:

Determination of photophysical and biochemical properties of mutated Broccoli−DFHBI-1T Complexes

The λ_{max}, relative brightness, dissociation constants and melting points were determined following the methods in reference 15 of the main text.

The emission was measured for solutions using “excess RNA” conditions, to ensure that no free fluorophore contributes to the fluorescence signal. The RNA concentration was 30 µM, while DFHBI-1T concentration was 2 µM. The fluorescence emission was determined in a Fluorolog-3 spectrofluorometer (Horiba Scientific) using the excitation wavelength commonly used for DFHBI-1T, 472 nm, with a side entrance and side exit slits of 3 nm. The integration time was 0.1 seconds, the emission was recorded from 482 nm to 700 nm, with 1 nm increments. The side entrance, front exit and side exit slits were 3 nm.

For the relative brightness, the fluorescence signal of Broccoli-DFHBI-1T complex was compared at different dilutions, using the U-Broccoli-DFHBI-1T as reference.

To calculate dissociation constant (K_D), we titrated increasing concentrations of DFHBI-1T into 50 nM of RNA. The fluorescence at 507 nm wavelength was determined in a Fluorolog-3 spectrofluorometer (Horiba Scientific), using the excitation wavelengths commonly used for DFHBI-1T (Excitation: 472 nm). The resulting data points were fitted to the Hill equation using Origin Pro Software.

To measure the thermostability of RNA-fluorophore complexes, 50 nM of RNA were incubated with 300 µM DFHBI-1T. Then fluorescence values were recorded in 5 °C increments from 10 °C to 85 °C, with a 2-min incubation at each temperature to allow for equilibration, using a CFX96 thermocycler (Bio-rad). The resulting data points were fitted to a biphasic model using Origin Pro Software.

Determination of A260/F507 ratio on dBroc mRNAs:

These methods are described in the Material and Methods section.

References:

1. Cavaluzzi, M.J., and Borer, P.N. (2004). Revised UV extinction coefficients for nucleoside-5′-monophosphates and unpaired DNA and RNA. Nucleic Acids Res *32*, e13. 10.1093/nar/gnh015.

2. Basanta-Sanchez, M., Temple, S., Ansari, S.A., D'Amico, A., and Agris, P.F. (2016). Attomole quantification and global profile of RNA modifications: Epitranscriptome of human neural stem cells. Nucleic Acids Research *44*, e26. 10.1093/nar/gkv971.

3. Yu, C.-T., and Allen, F.W. (1959). Studies of an isomer of uridine isolated from ribonucleic acids. Biochimica et Biophysica Acta *32*, 393–406. 10.1016/0006-3002(59)90612-2.

4. Davis, F.F., and Allen, F.W. (1957). RIBONUCLEIC ACIDS FROM YEAST WHICH CONTAIN A FIFTH NUCLEOTIDE. Journal of Biological Chemistry *227*, 907–915. 10.1016/S0021-9258(18)70770-9.

5. Shapiro, R., and Chambers, R.W. (1961). SYNTHESIS OF PSEUDOURIDINE. J. Am. Chem. Soc. *83*, 3920–3921. 10.1021/ja01479a057.

6. Michelson, A.M., and Cohn, W.E. (1962). Cyclo-pseudouridine and the Configuration of Pseudouridine. Biochemistry *1*, 490–495. 10.1021/bi00909a020.

7. Cohn, W.E. (1960). Pseudouridine, a Carbon-Carbon Linked Ribonucleoside in Ribonucleic Acids: Isolation, Structure, and Chemical Characteristics. Journal of Biological Chemistry *235*, 1488–1498. 10.1016/S0021-9258(18)69432-3.

8. Szer, W. (1965). Secondary structure of poly-5-methylcytidylic acid. Biochemical and Biophysical Research Communications *20*, 182–186. 10.1016/0006-291X(65)90343-8.

9. Martínez-Fernández, L., Pepino, A.J., Segarra-Martí, J., Banyasz, A., Garavelli, M., and Improta, R. (2016). Computing the Absorption and Emission Spectra of 5-Methylcytidine in Different Solvents: A Test-Case for Different Solvation Models. J. Chem. Theory Comput. *12*, 4430–4439. 10.1021/acs.jctc.6b00518.

10. Fox, J.J., Van Praag, D., Wempen, I., Doerr, I.L., Cheong, L., Knoll, J.E., Eidinoff, M.L., Bendich, A., and Brown, G.B. (1959). Thiation of Nucleosides. II. Synthesis of 5-Methyl-2'-deoxycytidine and Related Pyrimidine Nucleosides [1]. J. Am. Chem. Soc. *81*, 178–187. 10.1021/ja01510a042.

11. Ma, C., Cheng, C.C.-W., Chan, C.T.-L., Chan, R.C.-T., and Kwok, W.-M. (2015). Remarkable effects of solvent and substitution on the photo-dynamics of cytosine: a femtosecond broadband time-resolved fluorescence and transient absorption study. Phys. Chem. Chem. Phys. *17*, 19045–19057. 10.1039/C5CP02624E.

12. Shugar, D., and Fox, J.J. (1952). Spectrophotometric studies of nucleic acid derivatives and related compounds as a function of pH: I. Pyrimidines. Biochimica et Biophysica Acta *9*, 199–218. 10.1016/0006-3002(52)90147-9.

13. Sharonov, A., Gustavsson, T., Marguet, S., and Markovitsi, D. (2003). Photophysical properties of 5-methylcytidine. Photochem Photobiol Sci *2*, 362–364. 10.1039/b212664h.
